# Self-assembly of a helical zinc-europium complex: speciation in aqueous solution and luminescence

**DOI:** 10.3389/fchem.2013.00015

**Published:** 2013-09-11

**Authors:** Emmanuel Deiters, Svetlana V. Eliseeva, Jean-Claude G. Bünzli

**Affiliations:** ^1^Institute of Chemical Sciences and Engineering, École Polytechnique Fédérale de LausanneLausanne, Switzerland; ^2^Centre de Biophysique Moléculaire, Centre National de la Recherche Scientifique UPR 4301Orléans, France; ^3^Department of Advanced Materials Chemistry, WCU Center for Next Generation Photovoltaic Systems, Korea UniversitySejong-si, South Korea

**Keywords:** lanthanides, rare earths, self-assembly, dinuclear, bimetallic, stability constant, luminescence, helicates

## Abstract

Two new tridentate(NNO)-bidentate(NN) compartmental ligands, HL^5^ and HL^6^, are synthesized from pyridine and benzimidazole synthons. They react in aqueous solution under physiological conditions with Zn^II^, Ln^III^, or a mixture thereof, to yield complexes of different stoichiometries, 1:3, 2:2, 2:3, 1:1:3, the speciation of which is established by UV-visible titrations and ESI mass spectrometry. Photophysical studies of the Eu^III^-containing solutions in Tris-HCl 0.1 M (pH = 7.4) show that lanthanide luminescence arises from a unique N_6_O_3_ coordination site with pseudo *D*_3_ symmetry. Relevant parameters such as crystal field splitting, lifetime, radiative lifetime, and intrinsic quantum yield perfectly match those reported for dinuclear 4f-4f helicates in which the Eu^III^ ion has the same coordination environment.

## Introduction

**Scheme 1:**
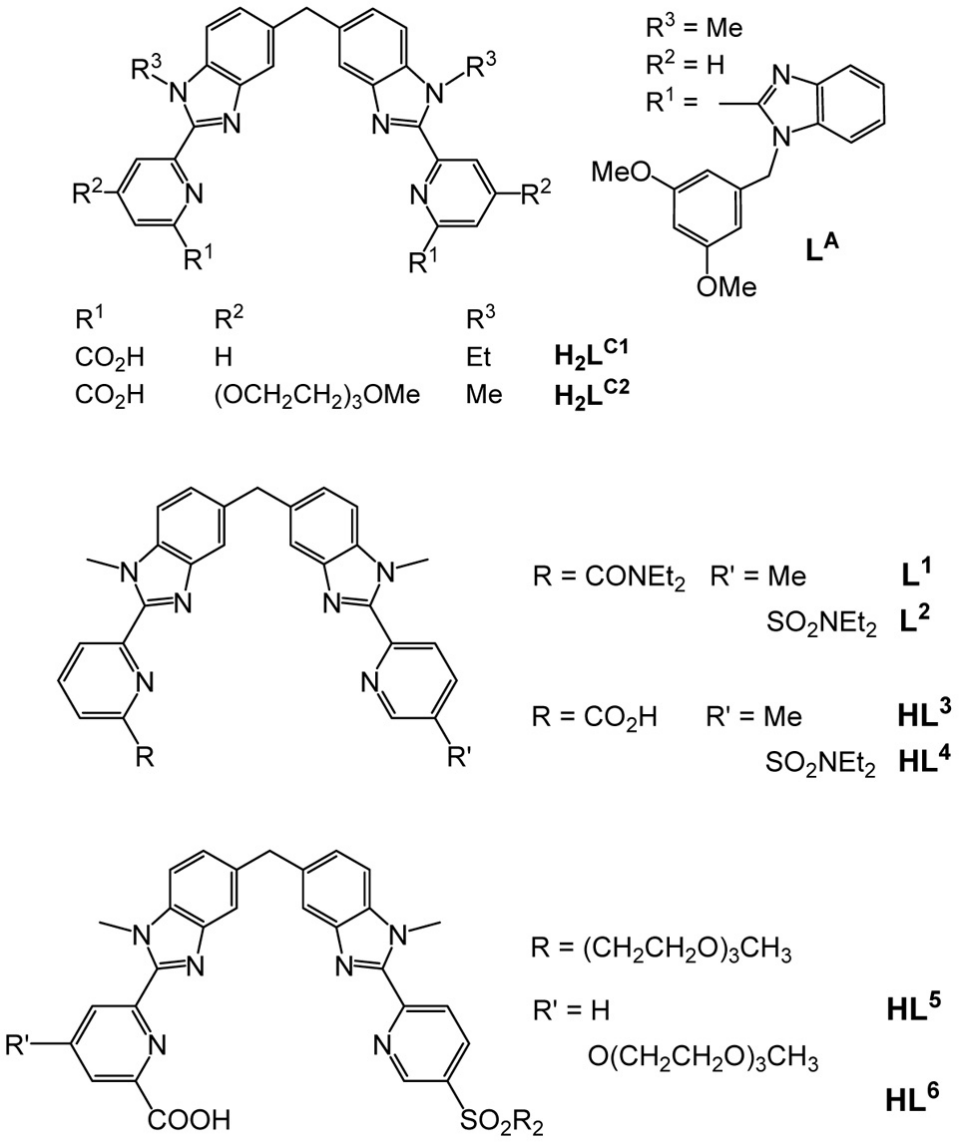
**Molecular structure of some ditopic ligands used for self-assembling dinuclear helicates**.

**Figure 1:**
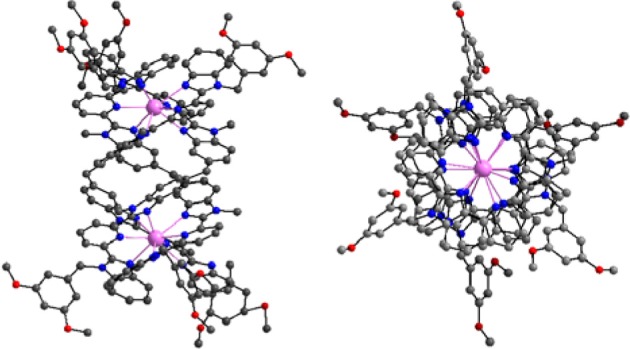
**Side and top views of the molecular structure of [Eu_2_(L^A^)_3_]^6+^.** Redrawn from data in Bernardinelli et al. (1992).

Helical structures have attracted chemists' attention when Linus Pauling published a seminal series of papers at the beginning of the 1950's dealing with the secondary structures of proteins induced by three-dimensional helical patterns (Pauling et al., 1951). The demonstration that helical structures can also be engineered at the molecular level by taking advantage of stereochemical properties of metal ions had to wait until 1987 when Jean-Marie Lehn isolated 3:2 Cu^I^:L double-stranded helical complexes that he named helicates, where L is an oligo-bipyridine ligand (Lehn et al., 1987). A few years later, Claude Piguet applied the same concept to trivalent lanthanide ions and successfully self-assembled the first Ln^III^ dinuclear triple-stranded helicate, [Eu_2_(L^A^)_3_]^6+^ (Scheme 1), the crystal structure of which evidences a stabilization of the molecular architecture by π−π stacking interactions between the ligand strands (Bernardinelli et al., 1992). The two 9-coordinate metal ions lie on a pseudo-*C*_3_ axis of symmetry (Figure 1); in solution the average symmetry of the edifice is *D*_3_ on NMR time scale (Piguet et al., 1993). This initial work paved the way for the development of several series of lanthanide polynuclear and polymetallic complexes including heterobimetallic nd-4f (Piguet et al., 1995a) and 4f-4f′ (André et al., 2004) chelates, as well as tri- and tetranuclear homometallic and heterometallic entities (Piguet et al., 2000; Piguet and Bünzli, 2010). Interestingly, the helicates are quite stable in solution despite large Coulomb repulsion between two neighboring cations which lie about 9 Å apart; careful thermodynamic considerations for 3d-4f and 4f-4f-4f helicates indeed show that the cation-cation repulsive energy (≈700 kJ·mol^−1^) is largely compensated by favorable solvation energy (Canard and Piguet, 2007). Furthermore, soluble helicates [Ln_2_(L^C1^)_3_] can be assembled in water and are highly stable, with logβ_23_ on the order of 26–30 (Elhabiri et al., 1998). Crystal structures revealed triple-stranded helicates with 9-coordinate metal ions (Ln = Eu, Tb) well-imbedded into the edifice and displaying interesting photophysical properties (Elhabiri et al., 1998; Gonçalves e Silva et al., 2002). Subsequent molecular engineering led to the series of the more water-soluble [Ln_2_(L^C2^)_3_] helicates and their bioconjugates which proved to be adequate luminescent bioprobes for live cell staining (Song et al., 2008; Chauvin et al., 2013) and for specific detection of biomarkers expressed by cancerous cells (Fernandez-Moreira et al., 2010).

One fascinating aspect of the polymetallic helical molecular edifices is the possibility of controlling the optical and/or magnetic properties of one ion by the other, through communication along the pseudo *C*_3_ axis. Examples are the tuning of the spin-crossover temperature in [FeLn(L^1,2^)_3_]^5+^ (Piguet et al., 1995b; Edder et al., 2000, 2001), (Scheme 1) or the lengthening of the excited state lifetimes of Nd^III^ and Yb^III^ in [CrLn(L^1^)_3_]^6+^ (Torelli et al., 2005). Such tunability would be of great help in the design of specific biosensors and stains, especially that [EuZn(L^2^)_3_]^5+^ proved to be quite luminescent in water with a quantum yield of 15% (Edder et al., 1997; Piguet and Bünzli, 2010). Bioprobes need to be water soluble and amenable to bioconjugation; unfortunately, helicates with the carboxylic acid derivatives HL^3^ and HL^4^ do not show enough water solubility for this purpose. In this paper, we apply to HL^3^ the successful strategy used in going from H_2_L^C1^ to H_2_L^C2^ in the hope of gaining access to luminescent and soluble 3d-4f helicates with ligands HL^5^ and HL^6^. More specifically, and as a first step toward engineering bioprobes based on such compounds, complex formation in the Eu^III^-Zn^II^-HL^6^ system and associated luminescent properties are investigated. Spectroscopically silent Zn^II^ has been chosen because it allows studying the coordination environment of the Eu^III^ ion without interference from the M^II^ ion.

## Results and discussion

### Ligand synthesis

**Scheme 2:**
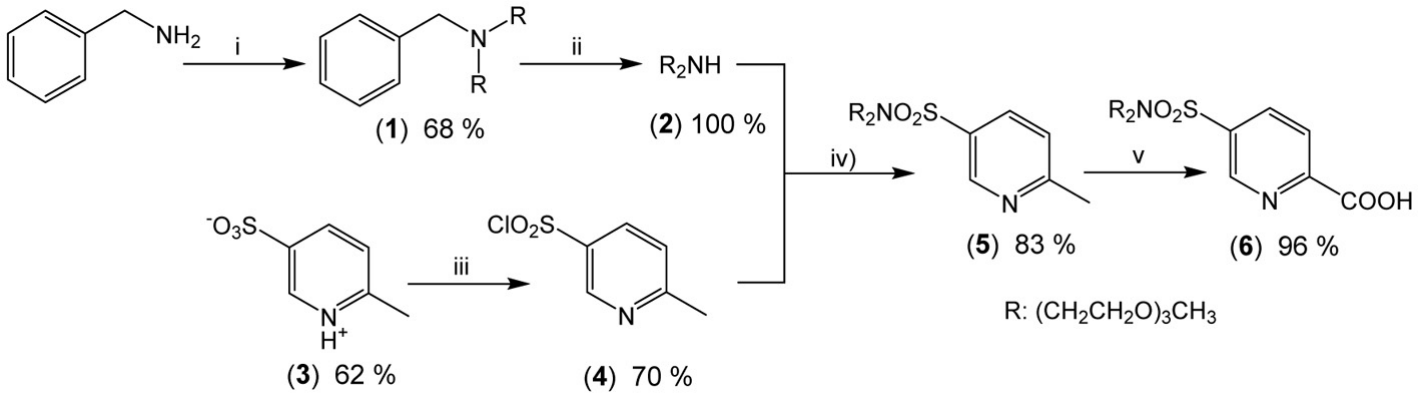
**Synthesis of the new precursor (6): (i) CH_3_(OCH_2_CH_2_)_3_Br (2.2 eqs), Na_2_CO_3_ (2.2 eqs), CH_3_CN (62°C, 76 h); (ii) Pd/C, H_2_ (70 bar, 45°C); (iii) PCl_5_ (1.5 eqs), POCl_3_ (reflux, 3 h); (iv) [CH_3_(OCH_2_CH_2_)_3_]_2_NH (1.2 eqs), NEt_3_ (5.0 eqs), CH_2_Cl_2_ (reflux, 16 h); (v) SeO_2_ (4.5 eqs); pyridine (reflux, 23 h)**.

**Scheme 3:**
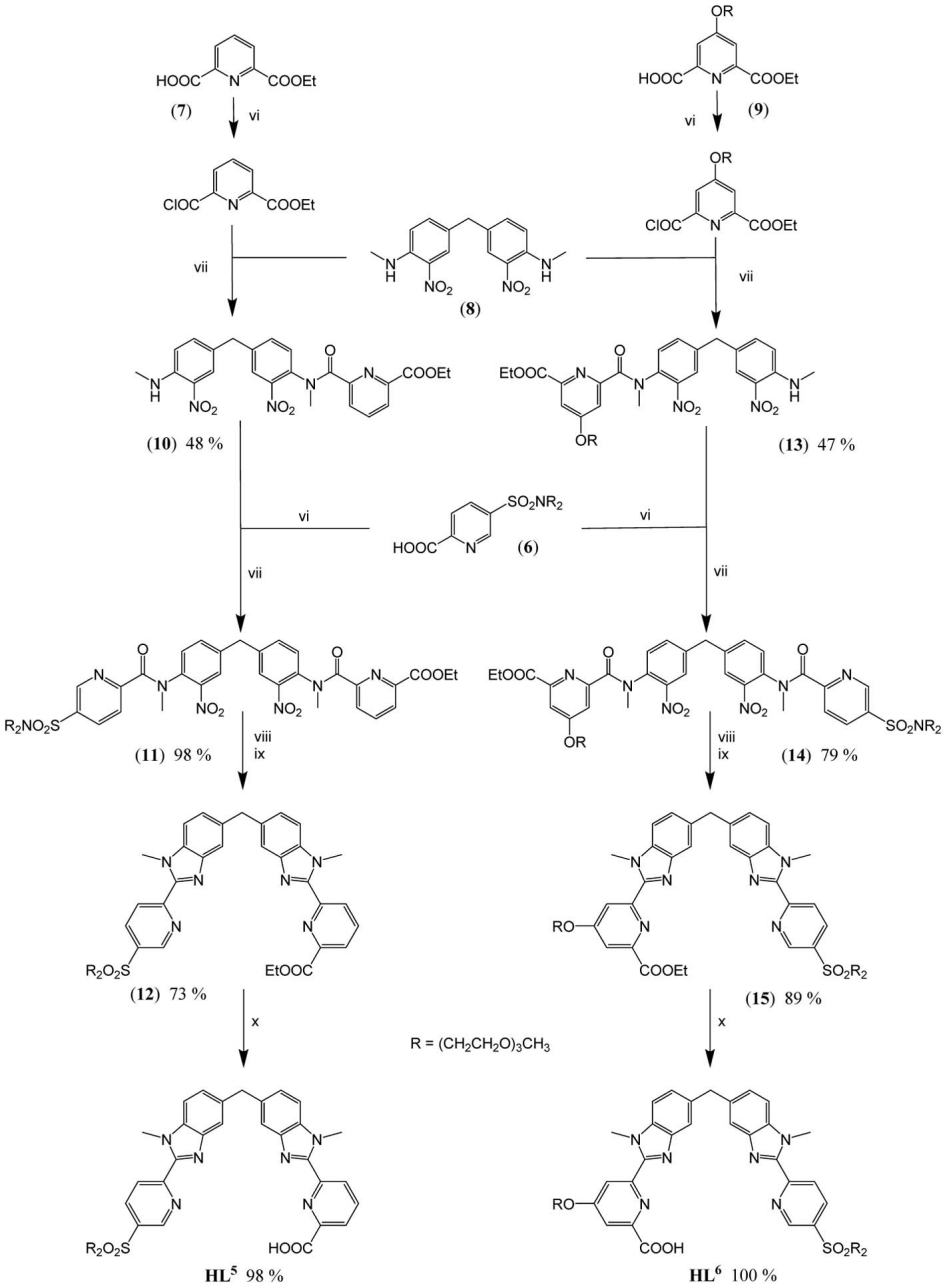
**Synthesis of HL^5^ and HL^6^: (vi) (6), (7), or (9) (1.0 eq.), SOCl_2_ (10 eqs), DMF (0.5 eq.), CH_2_Cl_2_ (reflux, 2 h); (vii) (8) (0.9 eq.), (10) (0.6 eq.) or (13) (0.4 eq.), NEt_3_, CH_2_Cl_2_ (reflux, 16 h); (viii) (11) or (14) (1.0 eq.), Fe (30 eqs), EtOH/H_2_O/HCl (reflux, 16 h); (ix) EtOH/H_2_SO_4_ (reflux, 4 h); (x) (12) or (15) (1.0 eq.), NaOH (1.2 eqs), EtOH/H_2_O (60°C, 16 h)**.

The underlying principle of the synthesis of HL^5^ and HL^6^ is the same as the one adopted for preparing ligands L^1, 2^ (Edder et al., 2000), and HL^3^ (Edder et al., 1997), namely a multistep strategy based on a modified Phillips reaction for the formation of the benzimidazole rings. However, the diethylamino groups are replaced by 2-[2-(2-methoxyethoxy)ethoxy]-*N*-{2-[2-(2-methoxyethoxy)ethoxy]ethyl}-ethanamino groups. Following our previous work (Deiters et al., 2009), the latter have been grafted on the key intermediate **(6)**, the synthesis of which is depicted on Scheme 2 while the routes for accessing ligands HL^5^ and HL^6^ are summarized on Scheme 3. Regarding sulfonation, the absence of directional electronic effects favoring electrophilic substitution in the ditopic ligands and the large number of carbon atoms amenable to sulfonation implies that the corresponding group has to be inserted in one of the starting building blocks, namely **(3)**.

Intermediate **(6)** was prepared in 7 steps and 23% yield from commercially available 2-picoline, benzylamine and triethylene glycol monomethyl ether (TEGOMe). The first two steps involve the synthesis of 2-[2-(2-methoxyethoxy)ethoxy]-*N*-{2-[2-(2-methoxyethoxy)-ethoxy]ethyl}ethanamine **(2)**: two TEGOMe arms are grafted on the primary benzylamine by reaction with BrTEGOMe in presence of a weak base (reaction i, Scheme 2) followed by selective and quantitative cleavage of the benzylamino group by continuous flow hydrogenation (reaction ii). Two other steps are necessary for preparing **(3)** from 2-picoline by treatment with oleum and subsequent oxidation with permanganate, according to a previously described procedure (Delarge, 1965). The sulfonate function of **(3)** is then activated by chlorination (reaction iii), in presence of a PCl_5_/POCl_3_ mixture to give the corresponding sulfonyl chloride **(4)**. Coupling between **(2)** and **(4)** is subsequently achieved under standard conditions (reaction iv) to yield sulfonamide **(5)**. Finally, oxidation of the methyl group of **(5)** into a carboxy group conducted in presence of an excess of SeO_2_ in refluxing pyridine affords synthon **(6)** in almost quantitative yield (reaction v).

Preparation of the segmental ligands HL^5,6^ is divided in fourth steps. Firstly, sequential Phillips coupling reactions (reactions vi/vii, Scheme 3) with successive introduction of different pyridine arms, **(6)**, **(7)**, and **(9)**, are performed on the central dinitro, bis-(*N*-methylamino)diphenylmethane synthon **(8)**. Secondly, the resulting bis-amides **(11)** and **(14)** are reduced (reactions viii, ix) in presence of a large excess of iron to form the *bis*(benzimidazole) intermediates **(12)** and **(15)**. Finally, basic hydrolysis of both ethylester functions (reactions x) leads to the targeted segmental ligands with overall yields (steps iii-x in Schemes 2, 3) of 18.8 and 18.4% for HL^5^ and HL^6^, respectively.

### Speciation in solution

Conditional stability constants of both homometallic (M = Zn^II^, La^III^, Eu^III^, Lu^III^) and heterometallic (M^1^ = Zn^II^, M^2^ = La^III^, Eu^III^, Lu^III^) complexes have been determined in Tris-HCl 0.1 M (pH 7.4) at 295 K by spectrophotometric titrations of the ligands (1.43 × 10^−5^ M for HL^5^ and 1.62 × 10^−5^ M for HL^6^, corresponding to an absorbance of about 0.5) with concentrated solutions of the metal perchlorates: 2.5–5.0 × 10^−3^ M for homometallic titrations and 2 × 10^−4^ M for each cation in the case of heterometallic titrations, in view of the poorer solubility of the hetero species. Titrations were performed batch wise for 20–25 [M]_*t*_/[HL^*i*^]_*t*_ (*i* = 5.6) ratios ranging between 0 and 2 (0 and 4 for Eu^III^ + Zn^II^, respectively). The first attempts to determine the speciation of the complexes revealed unreliable due to the slow kinetic of formation. Therefore, the time required to reach equilibrium was determined by luminescence spectroscopy, monitoring both the ligand fluorescence (Zn^II^-HL^6^ and Gd^III^-HL^6^ systems) and the phosphorescence of the Eu^III^ ion (Eu^III^-HL^6^ and Eu^III^-Zn^II^-HL^6^ systems). In the case of the lanthanide solutions with 1:3 and 2:3 Ln:HL^6^ ratios, steady states were reached within times not exceeding 1 h at room temperature. On the other hand, equilibrium times could be estimated at about 5–6 days at 313 K for heterometallic solutions. Consequently, solutions were carefully equilibrated before recording spectra, taking these data into consideration: 2 h at room temperature for homometallic solutions and 7 days at 313 K for heterometallic mixtures. These observations are interesting because in previous works, the kinetics of formation of homodinuclear 4f-4f helicates with a ligand similar to HL^C1^ but with carboxylic acid groups replaced with diethylamide groups (Hamacek et al., 2003) or with a bis(8-hydroxyquinolinate) ligand (Comby et al., 2009) was found to be fast in acetonitrile, equilibrium being reached within minutes. Similarly, [Eu_2_(L^C1^)_3_] forms within 10 min in water at pH 6.15 (Elhabiri et al., 2004). If the observed kinetics for the 1:3 and 2:3 Ln:HL^6^ complexes is not too different from the latter observation, formation of the Zn^II^Eu^III^ helicate is about two orders of magnitude slower.

In the case of ligand HL^5^, the spectra corresponding to the titration with lanthanum could be analyzed with a model including [La(L^5^)_*n*_]^(3 − *n*)+^ (*n* = 1, 2, 3) and [La_2_(L^5^)_3_]^3+^ species: the corresponding logβ_1*n*_ and logβ_23_ being 8.4(3), 14.2(5), 20.4(4), and 29.2(6), pointing to the formation of a very stable helicate, despite the mismatch between the ligand denticity (3 + 2) and the coordination number requirements of La^III^. On the other hand, data with lutetium gave a less satisfying fit. Moreover, when the ligand was titrated with zinc ions or with an equimolar mixture of zinc and lanthanide ions partial precipitation occurred and the residual absorbance at the end of the titration was markedly smaller than at the beginning. For this reason, no further experiments have been conducted with HL^5^ and we have concentrated our efforts on ligand HL^6^.

For the titrations of HL^6^ with Zn^II^ and Eu^III^, factor analysis pointed to the presence of 3–4 species in solution, including the free ligand. However, the fitting procedure was not straightforward and several models were tested. The best convergence and smallest residuals were obtained for the following sets of equilibria (charges are omitted for clarity):

(1) $$\text{Zn}+3{\text{L}}^{6}⇆[\text{Zn}{\left({\text{L}}^{6}\right)}_{3}]\text{ ​}\log\beta_{13}$$

(2) $$2\text{Zn}+2{\text{L}}^{6}+⇆[{\text{Zn}}_{2}{\left({\text{L}}^{6}\right)}_{2}]\;\log\beta_{22}$$

(3) $$2\text{Zn}+3{\text{L}}^{6}+⇆[{\text{Zn}}_{2}{\left({\text{L}}^{6}\right)}_{3}]\;\log\beta_{23}$$

(4) $$\text{Eu}+3{\text{L}}^{6}⇆[\text{Eu}{\left({\text{L}}^{6}\right)}_{3}]\;\log\beta_{13}$$

(5) $$2\text{Eu}+3{\text{L}}^{6}⇆[{\text{Eu}}_{2}{\left({\text{L}}^{6}\right)}_{3}]\;\log\beta_{23}$$

**Figure 2:**
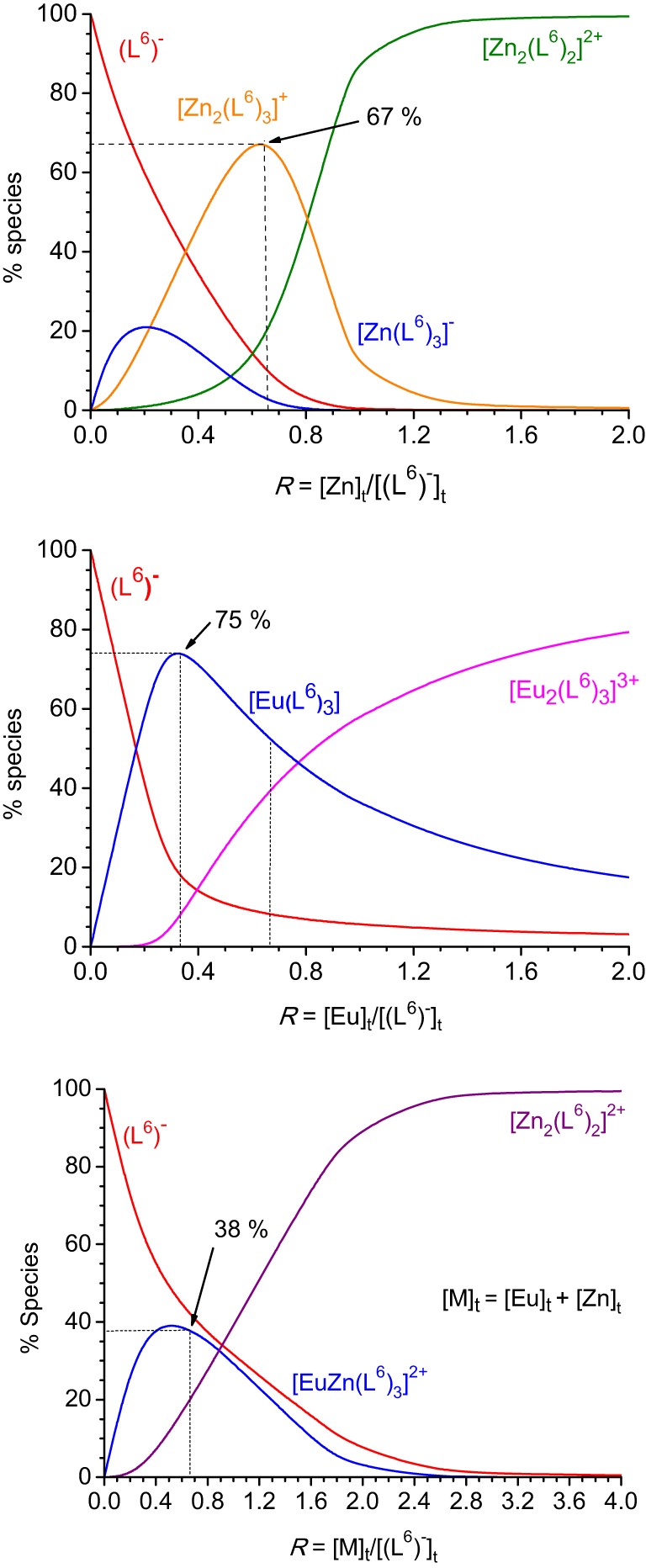
**Speciation diagrams calculated from the titration of HL^6^ 1.62 × 10^−5^ M in Tris-HCl (pH 7.4) at 295 K with zinc (top), europium (middle), and zinc + europium (1:1, bottom) perchlorates.** Corresponding stability constants are listed in Table 1.

Recalculated spectra are heavily correlated (Figures S1, S2, Supplementary Material), which explains the difficulties in the fitting procedure. The corresponding conditional stability constants are listed in Table 1. In the case of Zn^II^, the main species at 2:3 Zn:L stoichiometric ratio is the dinuclear complex (Figure 2, top) while the 1:3 complex remains a minor species (maximum speciation: 21% at *R* = 0.20); when *R* further increases, the 2:3 complex transforms into a 2:2 species. This behavior is in line with our previous results (Piguet and Bünzli, 2010). The tridentate-bidentate compartmental ligand HL^6^ is not well-suited for building triple-stranded helicates with Ln^III^ ions (two tridentate coordination units would be required) and this is seen in the corresponding speciation diagram: the dominant species is a 1:3 complex, with a 75% speciation for *R* = 0.33 (Figure 2, middle). For this ratio, only a small quantity of the 2:3 species is present (8%). Absorbance values extracted at different wavelengths for the titrations with Zn^II^ and Eu^III^ (Figures S1, S2, Supplementary Material) are compatible with the initial formation of 1:3 species.

**Table 1:** **Conditional stability constants in Tris-HCl (0.1 M, pH 7.4) and 295 K extracted from the spectrophotometric titrations of HL^6^**.

**Metal ion(s)**	**Logβ**_**13**_	**Logβ**_**22**_	**Logβ**_**23**_	**Logβ**_**113**_
Zn^II^	18.3 (4)	21.7 (4)	27.8 (4)	–
Eu^III^	17.5 (2)	–	22.8 (2)	–
Zn^II^ + Eu^III^ (1:1)	–	18.7 (3)	–	20.6 (3)

*Standard deviations are given within parentheses*.

The titration with a mixture of metal ions has been conducted in a slightly different way, due to reduced solubility of the formed products, the concentration of each metal ion has been set to 2.00 × 10^−4^ M only. Again fit of the data was difficult in view of the correlated spectra (Figure S3, Supplementary Material), so that the extracted data and corresponding discussion have to be taken with care. Indeed, logβ_22_ for [Zn_2_(L^6^)_2_]^2+^ extracted from this titration amounts to 18.7(3) whereas a value of 21.7(4) was found from the homometallic titration. We think, however, that the salient features are correct: contrary to what was expected, and found for other Zn^II^-Ln^III^ helicates in acetonitrile, for instance with L^2^ (Edder et al., 2000), the 1:1:3 species is not the dominant one, accounting for only 38% of the speciation at the 1:1:3 stoichiometric ratio. Another species is present in sizeable quantity (18%), namely the Zn^II^ 2:2 complex which is less stable than the 1:1:3 species by less than two orders of magnitude. So it seems there is competition between Zn^II^ and Eu^III^ for the tridentate coordination unit of (L^6^)^−^. This competition is further demonstrated by an experiment in which Zn^II^ was added to a 1:3 Eu:(L^6^)^−^stoichiometric solution 5.4 μM in Eu^III^ up to an Eu:Zn ratio equal to 1. The Eu^III^ luminescence intensity clearly decreases while ligand fluorescence centered at 450 nm increases (Figures S4, S5, Supplementary Material). Moreover, the ES-MS spectra discussed below point to other species being present in solution and the low solubility exhibited by this mixed system could well reflect the formation of polymeric (hydroxide?) species as well.

**Figure 3:**
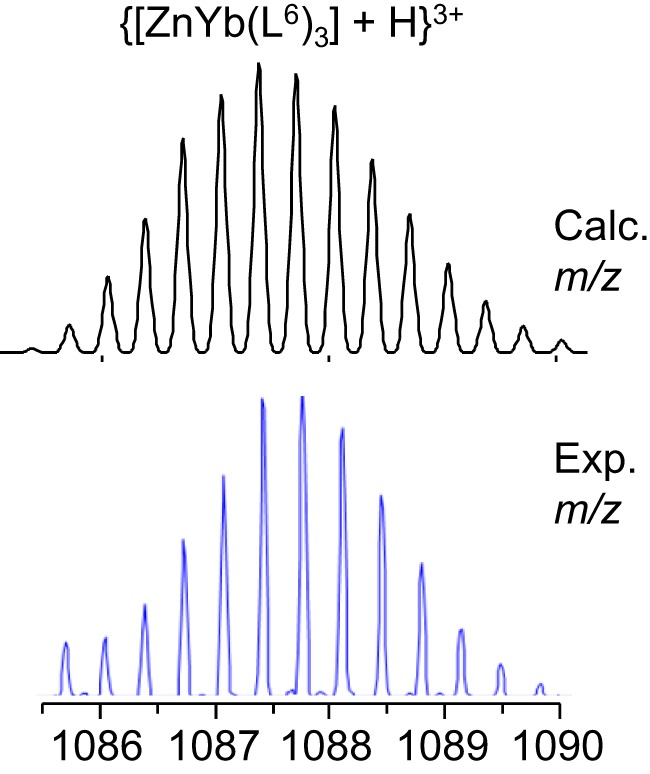
**Calculated (top) and experimental (bottom) isotopic distribution for {[ZnYb(L^6^)_3_] + H}^3+^**.

In order to substantiate the speciation determined by UV-visible titrations and, also, to determine if lighter and heavier lanthanides would lead to the same species in solution, ES-MS spectra of 1:1:3 Zn:Ln:(L^6^)^−^ solutions in acetonitrile:water (1:1) containing 1% of formic acid and with total ligand concentration equal to 3 mM have been recorded for Ln = Nd and Yb. The main observed peaks are listed in Table 2. The findings indeed partly corroborate those from UV-visible titrations. For the Nd-containing sample, the [ZnNd(L^6^)_3_]^2+^ complex is detected as a quadruple charged (+2H^+^) species, along with [Nd(L^6^)_3_] and [Zn(L^6^)_2_], the latter giving rise to several solvated species and/or adducts with sodium and potassium. Spectra for the Yb-containing samples are simpler; here again, the 1:1:3 species is detected through a peak with a sizeable intensity the high-resolution scan of which matches well the calculated isotopic distribution (Figure 3). As for neodymium, an ytterbium 1:3 species is present, as well as the 1:2 zinc complex. In both cases, no 2:2 zinc complex was identified though, contrary to UV-visible titration data; we note, however that the solvent is different and that the conditions in the spectrometer may lead to dissociation of this species.

**Table 2:** **Main peaks observed in the ESI-MS spectra of heterometallic solutions containing Zn^II^, Nd^III^ or Yb^III^, and (L^6^) in stoichiometric ratio 1:1:3 in acetonitrile/H_2_O/formic acid 49.5/49.5/1**.

**Solution**	**Species**	***m/z* (exp.)**	**Int.**	**Assignment**	***m/z* (calcd)**
Zn:Nd:(L^6^)^−^	[NdZn(L^6^)_3_]	808.53	20	[M + 2H]^4+^/4	808.53
1:1:3	[Nd(L^6^)_3_]	1594.58	6	[M + Na + H]^2+^/2	1594.59
		1604.57	5	[M + CH_3_CN + 2 H]^2+^/2	1604.61
	[Zn(L^6^)_2_]	1040.39	50	[M + 2 H]^2+^/2	1040.40
		1051.38	100	[M + Na + H]^2+^/2	1051.39
		1062.37	65	[M + CH_3_CN + 2 H]^2+^/2	1062.40
		701.25	65	[M + Na + 2 H]^2+^/3	701.26
		708.58	85	[M + 2 Na + H]^3+^/3	708.59
		713.90	85	[M + Na + K + H]^3+^/3	713.92
Zn:Yb:(L^6^)^−^	[YbZn(L^6^)_3_]	1087.73	25	[M + H]^3+^/3	1087.38
1:1:3	[Yb(L^6^)_3_]	1620.10	5	[M + 2 Na]^2+^/2	1620.10
	[Zn(L^6^)_2_]	1062.38	35	[M + 2 Na]^2+^/2	1062.38

### Photophysical properties of the solutions

**Figure 4:**
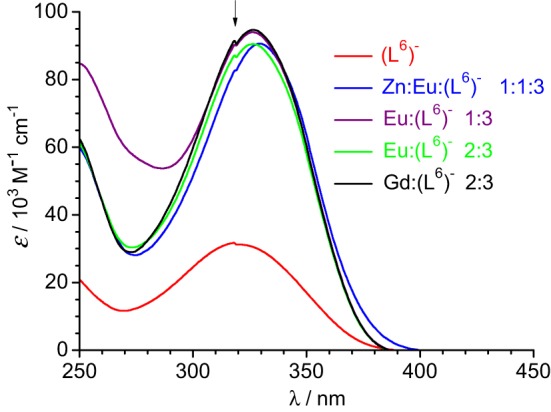
**Absorption spectra of ligand (L^6^)^−^ and solutions of it containing metal ions in Tris-HCl 0.1 M (pH 7.4).** Total ligand concentration: 5.4 μM for the free ligand and 16.2 μM for the other solutions. The arrow indicates an artifact due to lamp switching.

Absorption spectra of ligand (L^6^)^−^ and various solutions containing Ln^III^ ions (Ln = Eu, Gd) or an equimolar Eu^III^/Zn^II^ mixture are reported on Figure 4. The ligand absorption band at 319 nm can be assigned to a π→π^*^ transition involving intramolecular electron transfer from the benzimidazole units to the pyridine and carboxylic groups. This band is red-shifted to 326.5–327 nm in the solutions containing Ln^III^ ions only while the presence of Zn^II^ results in a slightly larger shift, to 329.5 nm. The molar absorption coefficients of the 1:3 solutions are, within experimental errors, equal to three times the molar absorption coefficient of the free ligand, while they are marginally smaller (−3.5%) for the 2:3 and 1:1:3 solutions (Table 3).

**Table 3:** **Photophysical data of the free and coordinated (L^6^)^−^ ligand at 298 K in Tris-HCl (pH 7.4). Energies are reported in cm^−1^**.

	**(L^**6**^)−**	**Eu:(L^**6**^)−**	**Gd:(L^**6**^)−**	**Eu:(L^**6**^)−**	**Zn:Eu:(L^**6**^)−**
		**1:3**	**1:3**	**2:3**	**1:1:3**
*E*(*π←π)^a^	31,450	30,630	30,580	30,630	30,350
Logε	4.50	4.97	4.98	4.96	4.96
*E*(^1^ππ*)^b^	21,450	22,350	22,350	22,450	21,950

a *From absorption spectra, maximum of band envelope*.

b *From fluorescence spectra, maximum of band envelope*.

**Figure 5:**
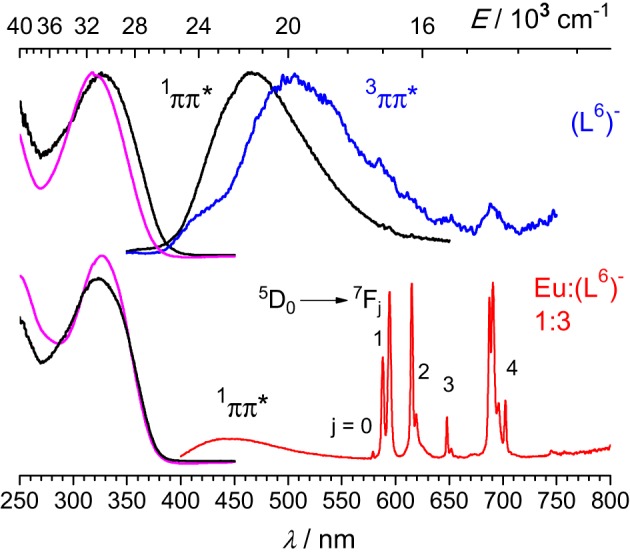
**Left: Normalized absorption and excitation spectra of the free ligand (top) and the 1:3 solution containing Eu^III^ (bottom). **Right:** Corresponding emission spectra upon excitation at 319 nm (**top**) or 326.5 nm (**bottom**).** Spectra are for solutions in Tris-HCl 0.1 M (pH 7.4) at 295 K, except for the phosphorescence spectrum of (L^6^)^−^ measured at 77 K with a 50-μs gate time.

Upon excitation into the 319-nm absorption band, ligand fluorescence emission is seen as a broad feature with maximum at 466 nm (Figure 5) and the corresponding excitation spectrum matches the absorption spectrum. At 77 K and upon enforcing a 50-μs delay time, weak phosphorescence is detected with a maximum at 506 nm. For the 1:3 Eu^III^ solution, fluorescence of the ligand is still seen, representing 37% of the total emission of the sample; this is consistent with the fact that the solution contains about 17% of free ligand (Figure 2, middle). In addition characteristic f-f emission from the Eu(^5^D_0_) level is detected. A striking feature is that this spectrum is quite typical of a species with pseudo *D*_3_ symmetry and is quasi identical to the one recorded for the [Eu_2_(L^C2^)_3_] helicate (Chauvin et al., 2008). In particular, the branching ratios expressed with respect to the intensity of the magnetic dipole transition, *I*(^5^D_0_ → ^7^F_*J*_)/*I*(^5^D_0_ → ^7^F_1_) are very similar for the two samples (data for [Eu_2_(L^C2^)_3_] are between parentheses): 0.02 (0.01), 1.00 (1.00), 0.87 (0.95), 0.16 (0.13), 1.62 (1.72), and 0.05 (n.a.). The splitting of the ^5^D_0_ → ^7^F_1_ transition is also very similar, 170 vs. 161 cm^−1^. These data point to luminescence arising from a coordination environment made up of 3 NNO moieties and very similar to the sites in [Eu_2_(L^C2^)_3_]; if some coordination were to occur through the bidentate site, then the coordination sphere would be completed by water molecules, leading to a poorly luminescent species. The solution also contains 8% of the 2:3 species, featuring two different metal ion sites (NNO)_3_ and (NN)_3_; the first one will give a spectrum identical to the one of the 1:3 complex, while the second will be poorly luminescent and therefore its contribution to the emission spectrum can be neglected. As an additional proof, the decay curve of the Eu(^5^D_0_) luminescence is perfectly monoexponential, with a lifetime of 2.7 ms (2.4 ms for [Eu_2_(L^C2^)_3_]), confirming that emission essentially originates from very similar coordination environments. Emission spectra of solutions with stoichiometric ratios Eu:(L^6^)^−^ 2:3 and Zn:Eu:(L^6^)^−^ 1:1:3 display spectra identical to the one of the 1:3 solution (Figure S6, Supplementary Material), consistent with the speciation reported in Figure 2; in particular, the emission intensity of the heterometallic solution is weak, due to the low concentration in 1:1:3 species (38%, which translates in 19% with respect to the europium site).

**Figure 6:**
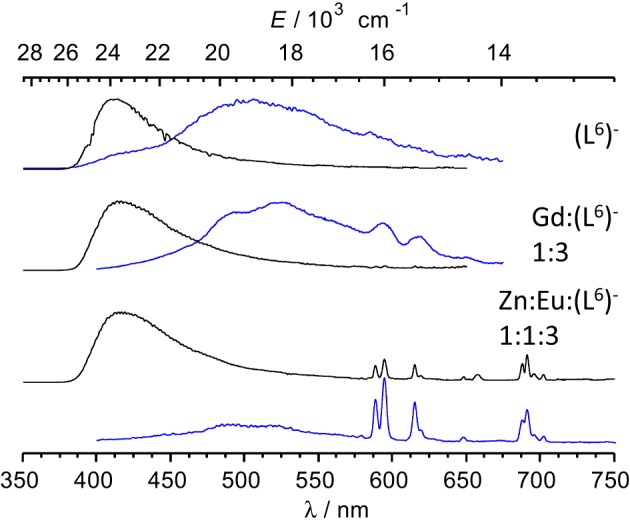
**Emission spectra of frozen solutions in Tris-HCl 0.1 M (pH 7.4, 77 K) of the ligand, a Gd^III^ sample and the Eu:Zn 1:1 sample; λ_exc_ = 3.9 (ligand) and 327 nm; [(L^6^)^−^] *t* = 16.2 μM; the Gd^III^ sample contains an Eu^III^ impurity.** Black lines: spectra recorded without time delay; blue lines: 50 μ s time delay.

Low-temperature emission spectra are presented on Figure 6 for the ligand, a Gd^III^-containing solution and the Eu:Zn 1:1 sample. Upon enforcing a 50-μs delay time, fluorescence of the ligand almost disappears to the benefit of a phosphorescence band centered at 509 nm. In the Gd:(L^6^)^−^ 1:3 sample, this band is red shifted at 525 nm and presents a vibrational structure (463, 494, 525, 559 nm) with ≈1200 cm^−1^ spacing, typical of a ring breathing mode. The Eu:Zn sample also displays ligand fluorescence and phosphorescence, again consistent with the speciation of the solution. The ligand phosphorescence band for this sample is identical to the one exhibited by the Gd sample. From these spectra, the 0-phonon energy of the triplet state of the bound ligand can be estimated to be 21,600 cm^−1^, while the 0-phonon energy of the singlet state lies at about 26,000 cm^−1^, as estimated from the onset of the fluorescence band. These energies are close to those reported for (L^C2^)^2−^ (Chauvin et al., 2008) and are adequate for ensuring efficient intersystem crossing and energy transfer onto the ^5^D_1_ (19,030 cm^−1^) and ^5^D_0_ (17,230 cm^−1^) levels of Eu^III^; on the other hand the quasi resonance between ^3^ππ^*^ and ^5^D_2_ (21,500 cm^−1^) may generate some back energy transfer.

Lifetimes and quantum yields of the Eu(^5^D_0_) level are reported in Table 4. Despite the different speciation of the solutions, the luminescence decays can be fitted with monoexponential functions and the resulting lifetimes lie within a narrow range, 2.46–2.69 ms, the shortest figure corresponding to the solution with the largest concentration of the 2:3 (or 1:1:3) species, in line with the somewhat shorter lifetime reported for [Eu_2_(L^Ci^)_3_], 2.43 ms for i = 1 (Elhabiri et al., 1999) and 2 (Chauvin et al., 2008) under similar experimental conditions. At low temperature, the lifetimes are much longer, pointing to temperature-dependent quenching mechanism(s) operating, e.g., back transfer (see above) or photo-induced electron transfer (PET). The radiative lifetimes can be estimated from the following equation:

(6) $$\tau_{\text{rad}}=\frac{1}{A_{\text{MD},\text{ 0}}\cdot n^{3}}\times \left(\frac{I_{\text{MD}}}{I_{\text{tot}}}\right)$$

where *A*_MD, 0_ = 14.65 s^−1^ is the decay rate of the magnetic dipole ^5^D_0_ → ^7^F_1_ transition, *n* is the refractive index of the medium, and *I*_MD_ and *I*_tot_ are the integrated emission areas of the ^5^D_0_ → ^7^F_1_ and ^5^D_0_ → ^7^F_*J*_ (*J* = 0–6) transitions. The corresponding data lead to evaluation of the intrinsic quantum yield, i.e., the quantum yield upon direct excitation onto the ^5^D_0_ level, since it is very difficult to determine experimentally in view of the faint molar absorption coefficients of the f-f transitions. The sensitization efficiency of the ligand can subsequently be calculated:

(7) $$Q_{\text{Eu}}^{\text{Eu}}=\frac{\tau_{\text{obs}}}{\tau_{\text{rad}}},\;\eta_{\text{sens}}=\frac{Q_{\text{Eu}}^{L}}{Q_{\text{Eu}}^{\text{Eu}}}$$

Both sets of data, radiative lifetimes and intrinsic quantum yields, are consistent for the three samples and, moreover they compare very well with data reported for the [Eu_2_(L^C1, 2^)_3_] helicates: τ_rad_ = 6.8−6.9 ± 0.3 ms and *Q*^Eu^_Eu_ = 36–37 ± 4% (Bünzli et al., 2008). On the other hand, absolute quantum yields and sensitization efficiencies are about three-fold smaller compared to the reference helicates (*Q*^*L*^_Eu_ = 21–24%, η_sens_ = 58–67%) due to the non-quantitative formation of the species. It is however noteworthy that the quantum yields of the two solutions containing Eu^III^ only, which feature approximately the same concentration of EuN_6_O_3_ sites (79 and 73% for 1:2 and 2:3 stoichiometric ratios, respectively), are equal, within experimental errors. For the heterometallic solution, the quantum yield is smaller, due to the small concentration of the 1:1:3 species, which, in addition features only one EuN_6_O_3_ coordination site.

**Table 4:** **Observed and radiative lifetimes (τ), intrinsic and absolute quantum yields (*Q*) of the Eu(^5^D_0_) level, as well as apparent ligand sensitization (η_sens_)for various samples in Tris-HCl 0.1 M (pH 7.4) with [(L^6^)^−^]_*t*_ = 16.2 μM, as determined at 298 K under ligand excitation (326–329 nm)**.

**Solution**	**Speciation^a^**	**τ_obs_/ms**	**τ_rad_/ms^b^**	**Q^Eu^_Eu_/%**	**Q^L^_Eu_/%**	**η_sens_/%**
Eu:(L^6^)^−^ 1:3	1:3 75%,	2.69 ± 0.02	7.5 ± 0.7	36 ± 4	8 ± 1	22 ± 4
	2:3 8%	3.56 ± 0.04^c^				
Eu:(L^6^)^−^ 2:3	1:3 53%,	2.46 ± 0.06	7.7 ± 0.8	32 ± 4	8 ± 1	25 ± 5
	2:3 39%					
Zn:Eu:(L^6^)^−^ 1:1:3	1:1:3 38%	2.54 ± 0.15	7.5 ± 0.7	34 ± 4	5 ± 1	15 ± 3
		3.15 ± 0.09^c^				

a *From Figure 2*.

b *Calculated with eq. (6)*.

c *In frozen solution at 77 K*.

## Conclusion

Reaction of ligands HL^5^ and HL^6^ (Scheme 3) under physiological conditions with equimolar quantities of Zn^II^ and Ln^III^ ions did not lead to a thermodynamically controlled assembly of the desired 3d-4f helicates, as expected from work with HL^4^, the 1:1:3 complex representing only 40% of the speciation. This can be traced back to stability of the Zn^II^ complexes with these ligands, even with respect to coordination to the tridentate unit, which is close to that of the Ln^III^ complexes. Attempts to isolate solid state samples of the helicates with transition metal ions such as Zn^II^, Cr^III^, Ru^II^ also afforded mixtures which we did not succeed to purify to an acceptable level. There is no doubt that the NNO moiety of the ligand should be remodeled to get better recognition of the Ln^III^ ions. An encouraging aspect, however, is that all luminescent data gathered for Eu^III^ solutions with different compositions point to the formation of either 1:3 or 2:3, or 1:1:3 complexes in which the lanthanide ion is coordinated to the tridentate chelating unit of ligand HL^6^, a judged by the crystal field splitting and other photophysical parameters which reflect the peculiar signature of the Eu(NNO)_3_ environment. In particular, the radiative lifetimes and intrinsic quantum yields match those of the previously reported helicates [Eu_2_(L^C1^)_3_] and [Eu_2_(L^C2^)_3_] with bis(tridentate) ligands.

On the other hand, the synthetic strategy applied for the preparation of tridentate-bidentate compartmental ligands aimed at assembling 3d-4f binuclear complexes proved to be valuable in that the ligands are obtained in reasonable yields given the number of steps needed. Moreover, the strategy can be adapted to graft other substituents on the sulfonate groups through modification of the key building block **6** (Scheme 2), so that this class of ligands represent a valuable addition to the chemistry of 3d-4f complexes.

## Materials and methods

### Synthesis of the ligands

Sulfonation of 2-picoline (Delarge, 1965) and bromination of TEGOMe (Deiters et al., 2009) have been previously reported so that these steps are not described here, except for NMR and ESI-MS characterization of **(3)**. Substituted pyridines **(7)** and (**9**) (Li et al., 2008; Deiters et al., 2009) and bis-(*N*-methylamino)diphenylmethane (**8**) (Piguet et al., 1992) were prepared according to literature procedures.

#### Starting materials and general procedures

Chemicals and solvents were purchased from Fluka A.G or Aldrich. Solvents were purified by passing them through activated alumina columns from Innovative Technology Inc. (Pangborn et al., 1996). Complexes were studied in solution only. Stock solutions of lanthanides were prepared just before use in freshly boiled, doubly distilled water from the corresponding Ln(ClO_4_)_3_·xH_2_O salts (Ln = La, Eu, Gd, Lu; *x* = 2.5–4.5). These salts were prepared from their oxides (Rhône-Poulenc, 99.99% and Catalysis or Research Chemicals, Phoenix, AZ) in the usual way (Bünzli and Mabillard, 1986). The concentrations of the solutions were determined by complexometric titrations using a standardized Na_2_H_2_EDTA in urotropine buffered medium and with xylenol orange as indicator (Schwarzenbach, 1957).

#### N-benzyl-2-[2-(2-methoxyethoxy)ethoxy]-N-{2-[2-(2-methoxyethoxy)ethoxy]ethyl}-ethanamine (1)

Benzylamine (2.5 g, 23.3 mmol), 1-bromo-2-[2-(2-methoxyethoxy)ethoxy]-ethane (11.59 g, 51.3 mmol) and Na_2_CO_3_ (5.44 g, 51.3 mmol) were heated and stirred in anhydrous CH_3_CN (30 mL) under inert atmosphere at 62°C for 76 h. After cooling, the reaction mixture was filtered and the white precipitate of Na_2_CO_3_ was washed with Et_2_O (about 50 mL). The resultant solution was evaporated under reduced pressure. The residue so obtained was re-dissolved in a hydrochloric acid solution (50 mL, 2 M) and extracted with Et_2_O (250 mL). The pH of the aqueous phase was then increased by addition of NaHCO_3_ up to saturation and the resulting solution was extracted with Et_2_O (3 × 250 mL). The three organic phases were combined, dried over Na_2_SO_4_, filtered, and concentrated under reduced pressure. The crude material was purified by column chromatography (silica gel, CH_2_Cl_2_/MeOH 100:0 → 97:3) to give pale yellow oil (6.32 g, 68% yield). ^1^H NMR (400 MHz, 298 K, CD_3_CN) δ (ppm): 2.66 (t, ^3^*J* = 6.2 Hz, 4H, NCH_2_CH_2_), 3.28 (s, 6H, OCH_3_), 3.44 (m, 4H, OCH_2_), 3.48–3.54 (m, 16H, OCH_2_), 3.67 (s, 2H, CH_2_), 7.24 (m. 1H, H_Ph._), 7.30 (m, 2H, H_Ph._), 7.33 (m, 2H, H_Ph._). ^13^C NMR (800 MHz, 298 K, CDCl_3_) δ (ppm): 53.86 (NCH_2_CH_2_O), 59.20 (OCH_3_), 59.86 (CH_2_), 70.00 (OCH_2_), 70.49 (OCH_2_), 70.68 (OCH_2_), 70.78 (OCH_2_), 72.06 (OCH_2_), 126.96 (CH_Ph._), 128.27 (CH_Ph._), 128.97 (CH_Ph_), 139.85 (C_Ph. quat._). ESI-MS *m*/*z* calcd for [M + H^+^] (found): 400.27 (400.04).

#### 2-[2-(2-methoxyethoxy)ethoxy]-N-{2-[2-(2-methoxyethoxy)ethoxy]ethyl}ethanamine (2)

Continuous flow hydrogenation of an ethanolic solution (0.05 M, 250 mL) of **(1)** (5.00 g, 12.5 mmol) was conducted with a safety H-Cube device from Thales Nanotechnology equipped with a HPLC pump under the following conditions; flow rate: 1 mL/min, catalyst cartridge: Pd/C; H_2_ pressure: 70 bar; temperature: 45°C. Ethanol and generated toluene were removed under reduced pressure and **(2)** obtained as a yellow oil was subsequently dried under vacuum (3.87 g, 100% yield). Note: one run was enough to fully convert **(1)** into **(2)** under the conditions mentioned above. ^1^H NMR (400 MHz, 298 K, CD_3_CN) δ (ppm): 2.73 (t, ^3^*J* = 6.2 Hz, 4H, NCH_2_CH_2_), 3.30 (s, 6H, OCH_3_), 3.46–3.57 (m, 20H, OCH_2_). ^13^C NMR (800 MHz, 298 K, CDCl_3_) δ (ppm): 49.30 (NCH_2_CH_2_), 59.21 (OCH_3_), 70.47 (OCH_2_), 70.63 (OCH_2_), 70.65 (OCH_2_), 70.67 (OCH_2_), 72.05 (OCH_2_). ESI-MS *m*/*z* calcd for [M + H^+^] (found): 310.23 (309.94).

#### 6-methylpyridinium-3-sulfonate (3)

This compound was synthetized according to a procedure described in the literature (Delarge, 1965); NMR and ESI-MS data are however reported here for the first time. ^1^H NMR (400 MHz, 298 K, D_2_O) δ (ppm): 2.81 (s, 3H, CH_3_), 7.97 (d, ^3^*J* = 8.5 Hz, 1H, H_Py._), 8.69 (d, ^3^*J* = 8.5 Hz, 1H, H_Py._), 8.97 (s, 1H, H_Py._) ^13^C NMR (800 MHz, 298 K, D_2_O) δ (ppm): 19.15 (CH_3_), 128.55 (CH_Py._), 138.35 (CH_Py._), 140.16 (C_Py. quat._), 142.95 (CH_Py._), 156.86 (C_Py. quat._). ESI-MS *m*/*z* calcd for [M+H^+^] (found): 174.02 (173.91); calcd for [M + CH_3_CN + H^+^] (found): 215.05 (214.87).

#### 6-methylpyridinium-3-sulfonyl chloride (4)

Synthon (**3**) (1.00 g, 5.78 mmol) and PCl_5_ (1.91 g, 9.25 mmol) were suspended in POCl_3_ (5 mL) and stirred at room temperature for 48 h. Then, POCl_3_ was rotor-evaporated and the residue was dissolved in CH_2_Cl_2_ (25 mL). Crushed ice (about 5 g) was slowly added to this solution *(beware, the reaction is highly exothermic!)* and the resulting phases were separated. The aqueous phase was extracted with CH_2_Cl_2_ (3 × 50 mL). The combined organic phases were combined, washed with dilute NaHCO_3_ solution, dried over Na_2_SO_4_, filtered and concentrated under reduced pressure. The crude material was charged and eluted on a chromatography column (silica gel, Et_2_O/petroleum ether 50:50 → 90:10) to give a white solid (0.766 g, 70% yield). ^1^H NMR (400 MHz, 298 K, CDCl_3_) δ (ppm): 2.73 (s, 3H, CH_3_), 7.42 (d, ^3^*J* = 8.5 Hz, 1H, H_Py._), 8.18 (dd, ^3^*J* = 8.5 Hz, ^4^*J* = 2.3 Hz, 1H, H_Py._), 9.12 (d, ^4^*J* = 2.3 Hz, 1H, H_Py._) ^13^C NMR (800 MHz, 298 K, DMSO-*d*_6_) δ (ppm): 19.36 (CH_3_), 127.68 (CH_Py._), 138.23 (CH_Py._), 142.49 (CH_Py._), 144.30 (C_Py. quat._), 154.30 (C_Py. quat._). ESI-MS *m*/*z* calcd for [M + H^+^] (found): 191.99 (191.81).

#### N,N-bis{2-[2-(2-methoxyethoxy)ethoxy]ethyl}-6-methylpyridine-3-sulfonamide (5)

Compound (**4**) (0.900 g, 4.71 mmol) was dissolved in dry CH_2_Cl_2_ (about 20 mL) under N_2_ steam and 3.3 mL NEt_3_ (23.6 mmol) was added. The resulting solution was refluxed and a solution of CH_2_Cl_2_ containing (**2**) (1.75 g, 5.66 mmol) was added dropwise. The reflux was subsequently maintained for 16 h. After cooling, the solvent and excess of NEt_3_ were rotor-evaporated under reduced pressure. The residue was dissolved in CH_2_Cl_2_ (50 mL) and a saturated aqueous solution of NH_4_Cl (50 mL) was added. After separation, the aqueous phase was extracted with 3 × 50 mL CH_2_Cl_2_. The combined organic phases were reduced to a volume of about 100 mL. This solution was dried over Na_2_SO_4_, filtered, and evaporated under reduced pressure. The crude material was purified by column chromatography (silica gel, CH_2_Cl_2_/MeOH 100:0 → 97:3) to afford amber oil (1.81 g, 83% yield). ^1^H NMR (400 MHz, 298 K, CD_3_CN) δ (ppm): 2.58 (s, 3H, CH_3_), 3.28 (s, 6H, OCH_3_), 3.40 (m, 4H, OCH_2_CH_2_), 3.44–3.57 (m, 20H, OCH_2_), 7.37 (d, ^3^*J* = 8.2 Hz, 1H, H_Py._), 8.03 (dd, ^3^*J* = 8.2 Hz, ^4^*J* = 2.4 Hz, 1H, H_Py._), 8.86 (d, ^4^*J* = 2.4 Hz, 1H, H_Py._) ^13^C NMR (800 MHz, 298 K, CDCl_3_) δ (ppm): 24.79 (CH_3_), 48.46 (NCH_2_CH_2_), 59.20 (OCH_3_), 69.90 (OCH_2_), 70.49 (OCH_2_), 70.62 (OCH_2_), 70.67 (OCH_2_), 72.04 (OCH_2_), 123.35 (CH_Py._), 134.04 (C_Py. quat._), 135.33 (CH_Py._), 147.71 (CH_Py._), 162.94 (C_Py. quat._). ESI-MS *m*/*z* calcd for [M + H^+^] (found): 465.23 (465.00); calcd for [M + Na^+^] (found): 487.01 (487.21).

#### 5-(bis{2-[2-(2-methoxyethoxy)ethoxy]ethyl}sulfamoyl)pyridine-2-carboxylic acid (6)

Compound (**5**) (1.80 g, 3.88 mmol) was added to a suspension of SeO_2_ (1.94 g, 17.45 mmol) in dry pyridine (60 mL) maintained under an N_2_ stream. The heterogeneous mixture was refluxed for 24 h and filtered through Celite® after cooling. Celite® was further washed with Et_2_O (about 100 mL) and the solvents were removed under reduced pressure. The residue was dissolved in distilled H_2_O (about 20 mL) and the pH was increased to 10 by addition of aqueous NaOH (5%). The aqueous phase was then extracted with CH_2_Cl_2_ (3 × 100 mL). The aqueous phase was acidified to pH 3 by adding aqueous hydrochloric acid (25%) and the resulting solution was extracted again with CH_2_Cl_2_ (3 × 100 mL). The organic phases were combined, reduced to a volume of about 100 mL, dried over Na_2_SO_4_, filtered, and rotor-evaporated under reduced pressure. After drying, **(6)** was obtained as an amber oil (1.83 g, 96% yield). ^1^H NMR (400 MHz, 298 K, acetone-*d*_6_) δ (ppm): 3.29 (s, 6H, OCH_3_), 3.45–3.49 (m, 12H, OCH_2_), 3.54 (m, 4H, OCH_2_), 3.58 (m, 4H, OCH_2_), 3.64 (m, 4H, OCH_2_), 8.32 (d, ^3^*J* = 8.1 Hz, 1H, H_Py._), 8.50 (dd, ^3^*J* = 8.1 Hz, ^4^*J* = 2.2 Hz, 1H, H_Py._), 9.09 (d, ^4^*J* = 2.2 Hz, 1H, H_Py._) ^13^C NMR (800 MHz, 298 K, CDCl_3_) δ (ppm): 47.71 (NCH_2_CH_2_), 59.16 (OCH_3_), 67.21 (OCH_2_), 69.37 (OCH_2_), 70.46 (OCH_2_), 70.57 (OCH_2_), 72.06 (OCH_2_), 123.97 (CH_Py._), 137.71 (CH_Py._), 141.33 (CH_Py._), 147.19 (CH_Py._), 148.35 (C_Py. quat._), 163.18 (COOH). ESI-MS *m*/*z* calcd for [M + H^+^] (found): 495.20 (495.00).

#### Ethyl 6-(methyl{4-[4-(methylamino)-3-nitrobenzyl]-2-nitrophenyl}carbamoyl)pyridine-2-carboxylate (10)

A mixture of 2,6-pyridinecarboxylic acid monoethyl ester (**7**) (1.00 g, 5.13 mmol), freshly distilled SOCl_2_ (6.10 g, 51.3 mmol), and dry DMF (200 μ L, 2.56 mmol) were refluxed in dry CH_2_Cl_2_ (50 mL) under an inert atmosphere for 2 h. After evaporation and pumping for 2 h, the pale yellow solid formed was re-dissolved in dry CH_2_Cl_2_ (50 mL). 3,3′-dinitro-4,4′-bis(*N*-methylamino)diphenylmethane (**8**) (1.46 g, 4.61 mmol) and NEt_3_ (2.00 mL) were added to this solution. The resulting mixture was refluxed for 16 h and evaporated. The red-brown residue was re-dissolved in CH_2_Cl_2_ (100 mL) and washed with half-saturated NH_4_Cl solution (100 mL). After separation, the aqueous phase was extracted with CH_2_CL_2_ (2 × 100 mL). The combined organic phases were dried over Na_2_SO_4_ and evaporated. The resulting red-brown solid was purified by column chromatography (silica gel, CH_2_Cl_2_/hexane 95:5 → CH_2_Cl_2_/MeOH 99:1) to give the mono-substituted product **(10)** as orange solid (1.09 g, 48% yield). ^1^H NMR (400 MHz, 433 K, DMSO-*d*_6_) δ (ppm): 1.30 (t, ^3^*J* = 7.2 Hz, 3H, OCH_2_CH_3_), 3.00 (d, ^3^*J* = 5.1 Hz, 3H, NHCH_3_), 3.41 (s, 3H, NCH_3_), 4.00 (s, 2H, CH_2_), 4.25 (q,^3^*J* = 7.2 Hz, 2H, OCH_2_CH_3_), 6.95 (d, ^3^*J* = 8.7 Hz, 1H, H_Benz._), 7.36 (d, ^3^*J* = 8.7 Hz, 1H, H_Benz._), 7.44 (d,^3^*J* = 7.8 Hz, 1H, H_Benz._), 7.51 (d, ^3^*J* = 7.8 Hz, 1H, H_Benz._), 7.69 (s (broad), 1H, NH), 7.83 (s, 1H, H_Benz._), 7.84 (d, ^3^*J* = 8.8 Hz, 1H, H_Py._), 7.89 (d, ^3^*J* = 9.2 Hz, 1H, H_Py._), 7.91 (s, 1H, H_Benz._), 7.98 (dd, ^3^*J* = 9.2 Hz, ^3^*J* = 8.8 Hz, 1H, H_Py._). ^13^C NMR (800 MHz, 327 K, DMSO-*d*_6_) δ (ppm): 13.66 (OCH_2_CH_3_), 29.43 (NHCH_3_), 37.47 (NCH_3_), 37.92 (CH_2_), 60.73 (OCH_2_CH_3_), 114.44 (CH_Benz._), 124.85 (CH_Benz._), 125.16 (CH_Benz._), 125.21 (CH_Py._), 126.34 (CH_Py._), 126.75 (CH_Py._), 130.59 (C_Benz. quat._), 131.10 C_Benz. quat._), 134.45 (CH_Benz._), 135.84 (C_Benz. quat._), 137.02 (CH_Benz._), 138.32 (CH_Py._), 141.95 (C_Benz. quat._), 144.60 (C_Benz. quat._), 145.03 (C_Benz. quat._), 145.62 (C_Py. quat._), 151.96 (C_Py. quat._), 163.57 (CONMe), 165.49 (COOEt). ESI-MS *m*/*z* calcd for [M + H^+^] (found): 494.17 (493.97); calcd for [M + Na^+^] (found): 516.14 (515.96).

Note: The mono- (**10**, **13**) and bis- (**11**, **14**) amide intermediates display at least two different conformations at 298 K with enough slow exchange rates to be observed on both ^1^H and ^13^C NMR spectra. Thus, the NMR spectra of these compounds were recorded at higher temperature to give one set of averaged signals.

#### Ethyl 6-[(4-{4-[{[5-(bis{2-[2-(2-methoxyethoxy)ethoxy]ethyl}sulfamoyl)pyridin-2-yl]-carbonyl}(methyl)amino]-3-nitrobenzyl}-2-nitrophenyl)(methyl)carbamoyl]pyridine-2-carboxylate (11)

A mixture of (**6**) (700 mg, 1.42 mmol), freshly distilled SOCl_2_ (3.37 g, 28.3 mmol), and dry DMF (55 μL, 0.708 mmol) were refluxed in dry CH_2_Cl_2_ (50 mL) under an inert atmosphere for 2 h. After evaporation and pumping for 2 h, the brown oil formed was re-dissolved in dry CH_2_Cl_2_ (25 mL) and NEt_3_ (2.00 mL) was added. This mixture was refluxed and a solution of dry CH_2_Cl_2_ (25 mL) containing (**10**) (437 mg, 8.85 mmol) was added drop-wise over a period of 30 min. The resulting solution was kept under reflux for 16 h. and evaporated. The brown residue was re-dissolved in CH_2_Cl_2_ (100 mL) and washed with half-saturated NH_4_Cl solution (100 mL). After separation, the aqueous phase was extracted with CH_2_Cl_2_ (2 × 100 mL). The combined organic phases were dried over Na_2_SO_4_ and evaporated. The crude material was purified by column chromatography (silica gel, CH_2_Cl_2_/MeOH 99:1 → CH_2_Cl_2_/MeOH 95:5) to afford brown oil (840 mg, 98% yield). ^1^H NMR (400 MHz, 433 K, DMSO-*d*_6_) δ (ppm): 1.29 (t, ^3^*J* = 7.2 Hz, 3H, OCH_2_CH_3_), 3.28 (s, 6H, OCH_3_), 3.40 (s, 3H, NCH_3_), 3.41 (s, 3H, NCH_3_), 3.41 (m, 4H, NCH_2_CH_2_), 3.46–3.61 (m, 20H, OCH_2_), 4.16 (s (broad), 2H, CH_2_), 4.25 (q, ^3^*J* = 7.2 Hz, 2H, OCH_2_CH_3_), 7.48 (d, ^3^*J* = 8.9 Hz, 2H, H_Benz._), 7.49 (d,^3^*J* = 8.9 Hz, 2H, H_Benz._), 7.80 (dd, ^3^*J* = 8.5 Hz, ^4^*J* = 1.0 Hz, 1H, H_Py._), 7.85 (dd, ^3^*J* = 7.9 Hz, ^4^*J* = 1.0 Hz, 1H, H_Py._), 7.86 (s, 1H, H_Benz._), 7.89 (d, ^3^*J* = 9.2 Hz, 1H, H_Py._), 7.90 (s, 1H, H_Benz._), 7.99 (dd, ^3^*J* = 8.5 Hz, ^3^*J* = 7.9 Hz, 1H, H_Py._), 8.23 (d, ^3^*J* = 9.2 Hz, 1H, H_Py._), 8.63 (s, 1H, H_Py._). ^13^C NMR (800 MHz, 327 K, DMSO-*d*_6_) δ (ppm): 13.69 (OCH_2_CH_3_), 37.45 (NCH_3_), 38.25 (NCH_3_), 47.24 (NCH_2_CH_2_), 47.37 (CH_2_), 57.82 (OCH_3_), 60.75 (OCH_2_CH_3_), 68.33 (OCH_2_), 68.48 (OCH_2_), 69.39 (OCH_2_), 69.42 (OCH_2_), 71.09 (OCH_2_), 123.89 (CH_Benz._), 124.75 (CH_Benz._), 124.82 (CH_Benz._), 125.15 (CH_Py._), 126.80 (CH_Py._), 131.21 (CH_Benz._), 134.39 (CH_Benz._), 134.54 (CH_Benz._), 135.21 (C_Benz. quat._), 135.77 (CH_Py._), 135.87 (CH_Py._), 136.19 (C_Benz. quat._), 136.68 (CH_Py._), 138.36 (CH_Py._), 140.55 (C_Benz. quat._), 141.37 (C_Benz. quat._), 145.02 (C_Benz. quat._), 145.61 (C_Py. quat._), 145.77 (C_Benz. quat._), 147.13 (C_Py. quat._), 151.86 (C_Py. quat._), 155.07 (C_Py. quat._), 163.61 (CONMe.), 165.25 (CONMe), 165.42 (COOEt). ESI-MS *m*/*z* calcd for [M + H^+^] (found): 970.36 (970.20); calcd for [M + Na^+^] (found): 992.34 (992.17).

#### Ethyl 6-[5-({2-[5-(bis{2-[2-(2-methoxyethoxy)ethoxy]ethyl}sulfamoyl)pyridin-2-yl]-1-methyl-1H-benzimidazol-5-yl}methyl)-1-methyl-1H-benzimidazol-2-yl]pyridine-2-carboxylate (12)

Freshly activated iron powder (1.41 g, 25.3 mmol) and HCl solution (1.75 mL, 25%) were added to an EtOH/H_2_O solution (165/41 mL) containing (**11**) (816 mg, 0.842 mmol). The mixture was refluxed under an inert atmosphere for 16 h. The solution was cooled, the excess of un-reacted iron filtered, and evaporated. The crude product was re-dissolved in absolute EtOH (30 mL). An H_2_SO_4_ solution (2 mL, 97%) was carefully added and the solution was refluxed overnight. It was cooled and the solvents were rotor-evaporated. Distilled water (100 mL) was added and the pH was adjusted to 6 with an aqueous saturated solution of NaHCO_3_. Na_2_EDTA (6.27 g, 16.8 mmol) was added to this solution followed by addition of H_2_O_2_ (1.5 mL, 30%) which resulted in the solution turning brown. The pH was then increased to 7 with an aqueous saturated solution of NaHCO_3_ before extraction with CH_2_Cl_2_ (5 × 100 mL). The organic phases were combined, dried over Na_2_SO_4_, filtered, and evaporated to dryness, resulting in a brown crude solid which was purified by column chromatography (silica gel; CH_2_Cl_2_ → CH_2_Cl_2_/MeOH 96:4) to give a pale yellow solid (532 mg, 79% yield). ^1^H NMR (400 MHz, 298 K, acetone-*d*_6_) δ (ppm): 1.43 (t, ^3^*J* = 6.8 Hz, 3H, OCH_2_CH_3_), 3.25 (s, 6H, OCH_3_), 3.44 (m, 4H, NCH_2_CH_2_), 3.50–3.54 (m, 12H, OCH_2_), 3.59 (m, 4H, OCH_2_), 3.66 (m, 4H, OCH_2_), 4.29 (s (broad), 2H, CH_2_), 4.35 (s, 3H, NCH_3_), 4.43 (s, 3H, NCH_3_), 4.44 (q, ^3^*J* = 6.8 Hz, 2H, OCH_2_CH_3_), 7.32 (dd, ^3^*J* = 8.5 Hz, ^4^*J* = 1.3 Hz, 1H, H_Benz._), 7.34 (dd, ^3^*J* = 8.5 Hz, ^4^*J* = 1.3 Hz, 1H, H_Benz._), 7.53 (d, ^3^*J* = 8.5 Hz, 1H, H_Benz._), 7.55 (d, ^3^*J* = 8.5 Hz, 1H, H_Benz._), 7.66 (d, ^4^*J* = 1.3 Hz, 1H, H_Benz._), 7.68 (d, ^4^*J* = 1.3 Hz, 1H, H_Benz._), 8.15 (d, ^3^*J* = 4.4 Hz, 2H, H_Py._), 8.42 (dd, ^3^*J* = 8.5 Hz, ^4^*J* = 2.4 Hz, 1H, H_Py._), 8.59 (d, ^3^*J* = 8.5 Hz, 1H, H_Py._), 8.62 (t, ^3^*J* = 4.4 Hz, 1H, H_Py._), 9.12 (d, ^4^*J* = 2.4 Hz, 1H, H_Py._). ^13^C NMR (800 MHz, 298 K, CDCl_3_) δ (ppm): 14.44 (OCH_2_CH_3_), 33.11 (NCH_3_), 33.27 (NCH_3_), 42.34 (CH_2_), 48.37 (NCH_2_CH_2_), 59.18 (OCH_3_), 62.01 (OCH_2_CH_3_), 69.79 (OCH_2_), 70.51 (OCH_2_), 70.62 (OCH_2_), 70.65 (OCH_2_), 72.01 (OCH_2_), 110.19 (CH_Benz._), 110.25 (CH_Benz._), 120.07 (CH_Benz._), 120.28 (CH_Benz._), 124.35 (CH_Benz._), 124.87 (CH_Py._), 125.32 (CH_Py._), 125.85 (CH_Py._), 127.48 (CH_Py._), 135.61 (CH_Py._), 136.09 (C_Benz. quat._), 136.25 (C_Benz. quat._), 136.61 (C_Benz. quat._), 137.04 (C_Benz. quat._), 138.00 (CH_Py._), 143.00 (C_Benz. quat._), 147.17 (C_Py. quat._), 147.21 (C_Py. quat._), 148.63 (C_Py. quat._), 149.27 (C_Benz. quat._), 150.80 (C_Py.quat._), 153.71 (C_Benz. quat._), 165.08 (COOEt). ESI-MS *m*/*z* calcd for [M + H^+^] (found): 874.38 (874.37); calcd for [M + 2H^+^]/2 (found): 437.83 (437.69).

#### 6-[5-({2-[5-(bis{2-[2-(2-methoxyethoxy)ethoxy]ethyl}sulfamoyl)pyridin-2-yl]-1-methyl-1H-benzimidazol-5-yl}methyl)-1-methyl-1H-benzimidazol-2-yl]pyridine-2-carboxylic acid (HL^4^)

Intermediate (**12**) (525 mg, 0.602 mmol) was dissolved in an absolute EtOH/H_2_O mixture (20:20 mL) containing NaOH (28.9 mg, 0.721 mmol). The mixture was stirred at 60°C for 16 h. After completion of the reaction, the solvents were evaporated. The residue was dissolved in distilled water (50 mL) and the resulting aqueous solution was acidified to pH = 2 by addition of 0.02 M hydrochloric acid. The acidic solution was then extracted with CH_2_Cl_2_ (5 × 100 mL), dried over Na_2_SO_4_ and evaporated. The crude product was triturated with hexane (100 mL), filtered, and dried under vacuum to give a pale yellow solid (498 mg, 98% yield). ^1^H NMR (400 MHz, 298 K, acetone-*d*_6_) δ (ppm): 3.25 (s, 6H, OCH_3_), 3.43 (m, 4H, NCH_2_CH_2_), 3.50–3.54 (m, 12H, OCH_2_), 3.59 (m, 4H, OCH_2_), 3.66 (m, 4H, OCH_2_), 4.30 (s (broad), 2H, CH_2_), 4.35 (s, 3H, NCH_3_), 4.38 (s, 3H, NCH_3_), 7.32 (d, ^3^*J* = 8.1 Hz, 1H, H_Benz._), 7.34 (d, ^3^*J* = 8.5 Hz, 1H, H_Benz._), 7.55 (d, ^3^*J* = 8.1 Hz, H_Benz._), 7.55 (d, ^3^*J* = 8.5 Hz, 1H, H_Benz._), 7.68 (s, 1H, H_Benz._), 7.68 (s, 1H, H_Benz._), 8.20 (d,^3^*J* = 6.4 Hz, 1H, H_Py._), 8.21 (d, ^3^*J* = 4.7 Hz, 1H, H_Py._), 8.41 (dd,^3^*J* = 8.5 Hz, ^4^*J* = 0.6 Hz, 1H, H_Py._), 8.60 (d,^3^*J* = 8.5 Hz, 1H, H_Py._), 8.60 (dd,^3^*J* = 6.4 Hz, ^3^*J* = 4.7 Hz, 1H, H_Py._), 9.12 (d,^4^*J* = 0.6 Hz, 1H, H_Py._). ^13^C NMR (800 MHz, 298 K, CDCl_3_) δ (ppm): 32.62 (NCH_3_), 33.31 (NCH_3_), 42.30 (CH_2_), 48.38 (NCH_2_CH_2_), 59.18 (OCH_3_), 69.79 (OCH_2_), 70.50 (OCH_2_), 70.62 (OCH_2_), 70.64 (OCH_2_), 72.01 (OCH_2_), 110.34 (CH_Benz._), 120.01 (CH_Benz._), 120.17 (CH_Benz._), 124.36 (CH_Benz._), 124.42 (CH_Benz._), 125.82 (CH_Py._), 125.86 (CH_Py._), 128.64 (CH_Py._), 135.51 (C_Benz. quat._), 135.69 (CH_Py._), 136.20 (C_Py. quat._), 136.95 (C_Benz. quat._), 137.31 (C_Benz. quat._), 139.24 (CH_Py._), 142.29 (C_Benz. quat._), 142.82 (C_Benz. quat._), 146.34 (C_Py. quat._), 147.20 (CH_Py._), 148.66 (C_Py. quat._), 148.71 (C_Py. quat._), 149.11 (C_Benz. quat._), 153.47 (C_Benz. quat._), 164.72 (COOH). ESI-MS *m*/*z* calc for [M + H^+^] (found): 846.35 (846.21); calcd for [M+2H^+^]/2 (found): 423.68 (423.75). Anal. Calcd for C_42_H_51_N_7_O_10_S·H_2_O (found): C, 58.43 (58.24); H, 6.18 (6.23); N, 11.36 (11.07).

#### Ethyl 4-{2-[2-(2-methoxyethoxy)ethoxy]ethoxy}-6-(methyl{4-[4-(methylamino)-3-nitrobenzyl]-2-nitrophenyl}carbamoyl)pyridine-2-carboxylate (13)

A mixture of 6-(ethoxycarbonyl)-4-{2-[2-(2-methoxyethoxy)ethoxy]ethoxy]pyridine-2-carboxylic acid **(9)** (0.718 g, 2.01 mmol), freshly distilled SOCl_2_ (2.39 g, 20.0 mmol), and dry DMF (77 μ L, 1.05 mmol) were refluxed in dry CH_2_Cl_2_ (25 mL) under an inert atmosphere for 2 h. After evaporation and pumping for 2 h, the pale yellow oil formed was re-dissolved in dry CH_2_Cl_2_ (25 mL). 3,3′-dinitro-4,4′-bis(*N*-methylamino)diphenylmethane (**8**) (635 mg, 2.01 mmol) andNEt_3_ (1.50 mL) were then added. The resulting mixture was refluxed for 16 h. and evaporated. The red-brown residue was re-dissolved in CH_2_Cl_2_ (100 mL) and washed with half-saturated NH_4_Cl solution (100 mL). After separation, the aqueous phase was extracted with CH_2_Cl_2_ (2 × 100 mL). The combined organic phases were dried over Na_2_SO_4_ and evaporated. The resulting red-brown solid was purified by column chromatography (silica gel, CH_2_Cl_2_ → CH_2_Cl_2_/MeOH 97:3) to give the mono-substituted product **(13)** as orange-red oil (0.614 g, 47% yield). ^1^H NMR (400 MHz, 433 K, DMSO-*d*_6_) δ (ppm): 1.27 (t, ^3^*J* = 7.2 Hz, 3H, OCH_2_CH_3_), 2.99 (d, ^3^*J* = 2.7 Hz, 3H, NHCH_3_), 3.27 (s, 3H, OCH_3_), 3.38 (s, 3H, NCH_3_), 3.45 (m, 2H, OCH_2_), 3.54–3.57 (m, 4H, OCH_2_), 3.60 (m, 2H, OCH_2_), 3.79 (m, 2H, OCH_2_), 3.99 (s, 2H, CH_2_), 4.21 (q, ^3^*J* = 7.2 Hz, 2H, OCH_2_CH_3_), 4.28 (m, 2H, OCH_2_), 6.95 (d, ^3^*J* = 8.9 Hz, 1H, H_Benz._), 7.36 (d,^4^*J* = 1.0 Hz, 1H, H_Py._), 7.37 (d, ^3^*J* = 8.9 Hz, 1H, H_Benz._), 7.42 (d,^4^*J* = 1.0 Hz, 1H, H_Py._), 7.43 (d,^3^*J* = 7.8 Hz, 1H, H_Benz._), 7.51 (d, ^3^*J* = 7.8 Hz, 1H, H_Benz._), 7.77 (s (broad), 1H, NH), 7.85 (s, 1H, H_Benz._), 7.92 (s, 1H, H_Benz._). ^13^C NMR (800 MHz, 327 K, DMSO-*d*_6_) δ (ppm): 13.33 (OCH_2_CH_3_), 30.13 (NHCH_3_), 38.17 (NCH_3_), 38.63 (CH_2_), 58.50 (OCH_3_), 61.47 (OCH_2_CH_3_), 68.70 (OCH_2_), 68.94 (OCH_2_), 70.11 (OCH_2_), 70.30 (OCH_2_), 70.52 (OCH_2_), 71.79 (OCH_2_), 112.53 (CH_Py._), 113.40 (CH_Py._), 115.15 (CH_Benz._), 125.54 (CH_Benz._), 125.91 (CH_Benz._), 127.05 (CH_Benz._), 131.28 (C_Benz. quat._), 131.71 C_Benz. quat._), 135.10 (CH_Benz._), 136.53 (C_Benz. quat._), 137.73 (CH_Benz._), 142.65 (C_Benz. quat._), 145.30 (C_Benz. quat._), 145.79 (C_Benz. quat._), 148.32 (C_Py. quat._), 154.47 (C_Py. quat._), 164.24 (CONMe), 166.07 (COOEt), 166.43 (C_Py.−O quat._). ESI-MS *m*/*z* calcd for [M + H^+^] (found): 656.26 (656.06).

#### Ethyl 6-[(4-{4-[{[5-(bis{2-[2-(2-methoxyethoxy)ethoxy]ethyl}sulfamoyl)pyridin-2-yl]-carbonyl}(methyl)amino]-3-nitrobenzyl}-2-nitrophenyl)(methyl)carbamoyl]-4-{2-[2-(2-methoxyethoxy)ethoxy]ethoxy}pyridine-2-carboxylate (14)

A mixture of (**6**) (1.12 g, 2.26 mmol), freshly distilled SOCl_2_ (5.38 g, 45.2 mmol), and dry DMF (87 μ L, 1.13 mmol) were refluxed in dry CH_2_Cl_2_ (50 mL) under an inert atmosphere for 2 h. After evaporation and pumping for 2 h, the brown oil formed was re-dissolved in dry CH_2_Cl_2_ (25 mL) and NEt_3_ (2.00 mL) was added. This mixture was refluxed and a solution of dry CH_2_Cl_2_ (25 mL) containing (**13**) (536 mg, 8.17 mmol) was added dropwise over a period of 30 min. The resulting solution was kept under reflux for 16 h. and evaporated. The brown residue was re-dissolved in CH_2_Cl_2_ (100 mL) and washed with half-saturated NH_4_Cl solution (100 mL). After separation, the aqueous phase was extracted with CH_2_Cl_2_ (2 × 100 mL). The combined organic phases were dried over Na_2_SO_4_ and evaporated. The crude material was purified by column chromatography (silica gel, CH_2_Cl_2_/MeOH 99:1 → CH_2_Cl_2_/MeOH 95:5) to afford brown oil (725 mg, 79% yield). ^1^H NMR (400 MHz, 433 K, DMSO-*d*_6_) δ (ppm): 1.28 (t, ^3^*J* = 6.8 Hz, 3H, OCH_2_CH_3_), 3.27 (s, 3H, OCH_3_), 3.28 (s, 6H, OCH_3_), 3.39 (s, 3H, NCH_3_), 3.40 (s, 3H, NCH_3_), 3.40 (m, 4H, NCH_2_CH_2_), 3.45–3.50 (m, 14H, OCH_2_), 3.51–3.58 (m, 12H, OCH_2_), 3.61 (m, 2H, OCH_2_), 3.80 (m, 2H, OCH_2_), 4.17 (s (broad), 2H, CH_2_), 4.23 (q, ^3^*J* = 6.8 Hz, 2H, OCH_2_CH_3_), 4.33 (m, 2H, OCH_2_), 7.38 (d, ^4^*J* = 2.4 Hz, 1H, H_Py._), 7.43 (d, ^4^*J* = 2.4 Hz, 2H, H_Py._), 7.45–7.52 (d, ^3^*J* = 8.2 Hz, 3H, H_Benz._), 7.80 (d, ^3^*J* = 8.2 Hz, 1H, H_Py._), 7.86 (s, 1H, H_Benz._), 7.91 (s, 1H, H_Benz._), 8.23 (dd, ^3^*J* = 8.2 Hz, ^4^*J* = 1.7 Hz, 1H, H_Py._), 8.64 (s, 1H, H_Py._). ^13^C NMR (800 MHz, 327 K, DMSO-*d*_6_) δ (ppm): 13.66 (OCH_2_CH_3_), 37.45 (NCH_3_), 38.27 (NCH_3_), 47.23 (NCH_2_CH_2_), 47.39 (CH_2_), 57.81 (OCH_3_), 60.79 (OCH_2_CH_3_), 68.03 (OCH_2_), 68.24 (OCH_2_), 68.33 (OCH_2_), 68.47 (OCH_2_), 69.39 (OCH_2_), 69.42 (OCH_2_), 69.57 (OCH_2_), 69.81 (OCH_2_), 71.09 (OCH_2_), 111.83 (CH_Py._), 112.76 (CH_Py._), 123.88 (CH_Benz._), 124.82 (CH_Benz._), 125.18 (CH_Py._), 131.12 (CH_Benz._), 131.19 (CH_Benz._), 134.38 (CH_Benz._), 134.50 (CH_Benz._), 135.20 (C_Benz. quat._), 135.76 (CH_Py._), 136.21 (C_Benz. quat._), 136.68 (CH_Py._), 140.50 (C_Benz. quat._), 141.39 (C_Benz. quat._), 145.03 (C_Benz. quat._), 145.77 (C_Py. quat._), 145.77 (C_Benz. quat._), 147.62 (C_Py. quat._), 153.67 (C_Py. quat._), 155.07 (C_Py. quat._), 163.58 (CONMe.), 165.30 (CONMe), 165.78 (COOEt), 166.50 (C_Py. −O quat._). ESI-MS *m*/*z* calcd for [M + H^+^] (found): 1132.44 (1132.28); calcd for [M + 2H^+^]/2 (found): 566.72 (566.76).

#### Ethyl 6-[5-({2-[5-(bis{2-[2-(2-methoxyethoxy)ethoxy]ethyl}sulfamoyl)pyridin-2-yl]-1-methyl-1H-benzimidazol-5-yl}methyl)-1-methyl-1H-benzimidazol-2-yl]-4-{2-[2-(2-methoxyethoxy)ethoxy]ethoxy}pyridine-2-carboxylate (15)

Freshly activated iron powder (1.07 g, 19.1 mmol) and HCl (1.35 mL, 25%) were added to an EtOH/H_2_O solution (125/31 mL) containing **(15)** (720 mg, 0.636 mmol). The mixture was refluxed under an inert atmosphere for 16 h. The solution was cooled, the excess of un-reacted iron filtered, and evaporated. The crude product was re-dissolved in absolute EtOH (30 mL); H_2_SO_4_ (2 mL, 97%) was added carefully and the solution was refluxed overnight. It was cooled and the solvents were evaporated. Distilled water (100 mL) was added and the pH was adjusted to 6 with an aqueous saturated solution of NaHCO_3_. Na_2_EDTA (4.74 g, 12.7 mmol) was added to this solution, followed by H_2_O_2_ (1.5 mL, 30%), which resulted in the solution turning brown. The pH was then increased to 7 with a saturated solution of aqueous NaHCO_3_ before extraction with CH_2_Cl_2_ (5 × 100 mL). The organic phases were combined, dried over Na_2_SO_4_, filtered, and evaporated to dryness, resulting in a brown crude solid which was purified by column chromatography (silica gel; CH_2_Cl_2_ → CH_2_Cl_2_/MeOH 95:5) to give a pale yellow solid (585 mg, 89% yield). ^1^H NMR (400 MHz, 298 K, acetone-*d*_6_) δ (ppm): 1.42 (t, ^3^*J* = 7.2 Hz, 3H, OCH_2_CH_3_), 3.25 (s, 3H, OCH_3_), 3.26 (s, 6H, OCH_3_), 3.43 (m, 4H, NCH_2_CH_2_), 3.44 (m, 2H, OCH_2_), 3.50–3.55 (m, 12H, OCH_2_), 3.56–3.62 (m, 8H, OCH_2_), 3.65–3.70 (m, 6H, OCH_2_), 3.93 (m, 2H, OCH_2_), 4.29 (s (broad), 2H, CH_2_), 4.35 (s, 3H, NCH_3_), 4.41 (s, 3H, NCH_3_), 4.42 (q, ^3^*J* = 6.8 Hz, 2H, OCH_2_CH_3_), 4.45 (m, 2H, OCH_2_), 7.31 (dd, ^3^*J* = 8.5 Hz, ^4^*J* = 1.4 Hz, 1H, H_Benz._), 7.35 (dd,^3^*J* = 8.8 Hz, ^4^*J* = 1.4 Hz, 1H, H_Benz._), 7.53 (d,^3^*J* = 8.5 Hz, 1H, H_Benz._), 7.55 (d, ^3^*J* = 8.8 Hz, 1H, H_Benz._), 7.65 (d, ^4^*J* = 1.4 Hz, 1H, H_Benz._), 7.68 (d, ^4^*J* = 1.4 Hz, 1H, H_Benz._), 7.68 (d, ^4^*J* = 2.4 Hz, 1H, H_Py._), 8.14 (d, ^4^*J* = 2.4 Hz, 1H, H_Py._), 8.40 (dd, ^3^*J* = 8.5 Hz, ^4^*J* = 2.4 Hz, 1H, H_Py._), 8.60 (d, ^3^*J* = 8.5 Hz, 1H, H_Py._), 9.12 (d, ^4^*J* = 2.4 Hz, 1H, H_Py._). ^13^C NMR (800 MHz, 298 K, CDCl_3_) δ (ppm): 14.42 (OCH_2_CH_3_), 33.12 (NCH_3_), 33.26 (NCH_3_), 42.36 (CH_2_), 48.37 (NCH_2_CH_2_), 59.18 (OCH_3_), 59.21 (OCH_3_), 62.01 (OCH_2_CH_3_), 68.34 (OCH_2_), 69.35 (OCH_2_), 69.80 (OCH_2_), 70.51 (OCH_2_), 70.62 (OCH_2_), 70.65 (OCH_2_), 70.75 (OCH_2_), 70.79 (OCH_2_), 71.09 (OCH_2_), 72.02 (OCH_2_), 70.05 (OCH_2_), 110.19 (CH_Benz._), 110.23 (CH_Benz._), 111.93 (CH_Py._), 113.53 (CH_Py._) 120.01 (CH_Benz._), 120.29 (CH_Benz._), 124.34 (CH_Benz._), 125.22 (CH_Py._), 125.85 (CH_Py._), 135.61 (CH_Py._), 136.08 (C_Benz. quat._), 136.25 (C_Benz. quat._), 136.53 (C_Benz. quat._), 137.06 (C_Benz. quat._), 142.76 (C_Py. quat._), 143.01 (C_Benz. quat._), 147.17 (C_Py. quat._), 148.64 (C_Py. quat._), 148.75 (C_Py. quat._), 149.49 (C_Benz. quat._), 152.42 (C_Benz. quat._), 153.71 (C_Benz. quat._), 165.05 (COOEt), 166.50 (C_Py.−O quat._). ESI-MS *m*/*z* calcd for [M + H^+^] (found): 1036.47 (1036.25); calcd for [M + 2H^+^]/2 (found): 518.74 (518.86).

#### 6-[5-({2-[5-(bis{2-[2-(2-methoxyethoxy)ethoxy]ethyl}sulfamoyl)pyridin-2-yl]-1-methyl-1H-benzimidazol-5-yl}methyl)-1-methyl-1H-benzimidazol-2-yl]-4-{2-[2-(2-methoxyethoxy)ethoxy]ethoxy}pyridine-2-carboxylic acid (HL^5^)

Intermediate (**16**) (575 mg, 5.56 × 10^−1^ mmol) was dissolved in an absolute EtOH/H_2_O solution (20/5 mL) containing NaOH (26.7 mg, 0.667 mmol). This mixture was stirred at 60°C for 16 h. After completion of the reaction, the solvents were evaporated. The residue was dissolved in distilled water (50 mL) and the resulting aqueous solution was acidified to pH = 2 by addition of 0.02 M hydrochloric acid. The acidic solution was then extracted with CH_2_Cl_2_ (5 × 100 mL), dried over Na_2_SO_4_, and evaporated. The crude product was triturated with hexane (100 mL), filtered and dried under vacuum to give a pale yellow solid (560 mg, 100% yield). ^1^H NMR (400 MHz, 298 K, acetone-*d*_6_) δ (ppm): 3.25 (s, 9H, OCH_3_), 3.43 (m, 4H, NCH_2_CH_2_), 3.45 (m, 2H, OCH_2_), 3.50–3.54 (m, 12H, OCH_2_), 3.55–3.62 (m, 8H, OCH_2_), 3.65–3.70 (m, 6H, OCH_2_), 3.93 (m, 2H, OCH_2_), 4.29 (s (broad), 2H, CH_2_), 4.33 (s, 3H, NCH_3_), 4.35 (s, 3H, NCH_3_), 4.47 (m, 2H, OCH_2_), 7.31 (dd, ^3^*J* = 8.5 Hz, ^4^*J* = 1.4 Hz, 1H, H_Benz._), 7.34 (dd, ^3^*J* = 8.5 Hz, ^4^*J* = 1.4 Hz, 1H, H_Benz._), 7.53 (d, ^3^*J* = 8.5 Hz, 1H, H_Benz._), 7.55 (d, ^3^*J* = 8.5 Hz, 1H, H_Benz._), 7.65 (d, ^4^*J* = 1.4 Hz, 1H, H_Benz._), 7.68 (d, ^4^*J* = 1.4 Hz, 1H, H_Benz._), 7.68 (d, ^4^*J* = 2.4 Hz, 1H, H_Py._), 8.14 (d, ^4^*J* = 2.4 Hz, 1H, H_Py._), 8.40 (dd, ^3^*J* = 8.2 Hz, ^4^*J* = 2.4 Hz, 1H, H_Py._), 8.60 (d, ^3^*J* = 8.2 Hz, 1H, H_Py._), 9.12 (d, ^4^*J* = 2.4 Hz, 1H, H_Py._). ^13^C NMR (800 MHz, 298 K, CDCl_3_) δ (ppm): 32.80 (NCH_3_), 33.29 (NCH_3_), 42.33 (CH_2_), 48.39 (NCH_2_CH_2_), 59.18 (OCH_3_), 59.20 (OCH_3_), 68.67 (OCH_2_), 69.24 (OCH_2_), 69.80 (OCH_2_), 70.51 (OCH_2_), 70.62 (OCH_2_), 70.65 (OCH_2_), 70.74 (OCH_2_), 70.78 (OCH_2_), 71.11 (OCH_2_), 72.02 (OCH_2_), 72.03 (OCH_2_), 110.27 (CH_Benz._), 110.31 (CH_Benz._), 111.38 (CH_Py._), 113.85 (CH_Py._), 120.02 (CH_Benz._), 120.23 (CH_Benz._), 124.35 (CH_Benz._), 125.58 (CH_Py._), 125.86 (CH_Py._), 135.64 (C_Benz. quat._), 135.71 (CH_Py._), 136.12 (C_Py. quat._), 136.25 (CH_Py._), 136.98 (C_Benz. quat._), 139.42 (C_Benz. quat._), 142.44 (C_Benz. quat._), 142.95 (C_Benz. quat._), 147.18 (C_Py. quat._), 148.68 (C_Py. quat._), 149.12 (C_Benz. quat._), 150.54 (C_Py. quat._), 153.62 (C_Benz. quat._), 164.90 (COOH), 167.29 (C_Py−O quat._). ESI-MS *m*/*z* calcd for [M + H^+^] (found): 1008.44 (1008.29); calcd for [M + 2H^+^]/2 (found): 504.72 (504.87). Anal. Calcd for C_49_H_65_N_7_O_14_S·H_2_O (found): C, 57.39 (57.14); H, 6.58 (6.65); N, 9.56 (9.29).

## Analytical and spectroscopic measurements

Elemental analyses were performed by Dr. E. Solari, Elementary Analysis Laboratory of the Institute of Chemical Sciences and Engineering, EPFL. NMR spectra were measured on Bruker Avance DRX 400 (^1^H, 400 MHz), AV 600 (^13^C, 150.864 MHz), and AV 800 (^13^C, 201.54 MHz) spectrometers. Spectra of organic compounds were recorded in CDCl_3_ (99.8%), CD_3_CN (99.8%), acetone-*d*_6_ (99.5%), DMSO-*d*_6_ (99.8%), and D_2_O (99.9%), all from Aldrich Chemicals. Deuterated solvents were taken as internal standards; chemical shift values are given in ppm with respect to TMS and *J* values are reported in Hz. The ESI-MS spectra of the organic compounds were obtained on a Finningan TSQ 7100 spectrometer using 10^−5^–10^−4^ M solutions in acetonitrile/H_2_O/formic acid (49.5/49.5/1) or MeOH; the capillary temperature was set to 180°C and the ion spray voltage to 3.5 kV. The instrument was calibrated using horse myoglobin and the analyses were conducted in positive mode. Phosphoric acid was used for mass calibration in the range 500–2000 *m*/*z*. Data were acquired and processed with Masslynx version 4.0. Electrospray conditions were as follows: capillary voltage, 3 kV; source temperature, 80°C; cone voltage, 35 V; source block temperature, 150°C. The ESI nebulization and drying gas was nitrogen. The sample was introduced through a syringe pump operating at 20 μ L·min^−1^. Simulation of spectra was achieved with Molecular Weight Calculator 6.42®. UV/Vis absorption spectra were measured in 1.0 cm quartz Suprasil® cells on a Perkin-Elmer Lambda 900 spectrometer. Stability constants were determined by spectrophotometric titration of (L^4,5^)^−^ by Eu^III^ or Eu^III^/Zn^II^ (1:1) in Tris-HCl 0.1 M (pH 7.4) under N_2_ atmosphere. All titrations were performed batch wise in thermostated (25.0 ± 0.1°C) 1-cm quartz cuvettes. Factor analysis (Malinowski and Howery, 1991) and mathematical treatment of the spectrophotometric data were performed with the Specfit® software (Gampp et al., 1986). Luminescence spectra and lifetimes were collected either on a Horiba-Jobin Yvon FL 3-22 fluorometer or on a home-made high-resolution set-up, according to procedures published previously (Rodriguez-Cortinas et al., 2002). Quantum yields were measured by an absolute method using a specially designed integration sphere (Aebischer et al., 2009).

### Conflict of interest statement

The authors declare that the research was conducted in the absence of any commercial or financial relationships that could be construed as a potential conflict of interest.

## Supplementary material

Figure S1 **(Top) Re-calculated spectra from the titration of HL^6^ with zinc perchlorate at 295 K and pH 7.4. (Bottom) Absorbance values extracted at different wavelengths during the titration compared with theoretical prediction from the stability extracted from the fit procedure (Table 1)**.

Figure S2 **(Top) Re-calculated spectra from the titration of HL^6^ with europium perchlorate at 295 K and pH 7.4. (Bottom) Absorbance values extracted at different wavelengths during the titration compared with theoretical prediction from the stability extracted from the fit procedure (Table 1)**.

Figure S3 **(Top) Re-calculated spectra from the titration of HL^6^ with zinc and europium perchlorate (1:1) at 295 K and pH 7.4. (Bottom) Absorbance values extracted at different wavelengths during the titration compared with theoretical prediction from the stability extracted from the fit procedure (Table 1)**.

Figure S4 **Effect on luminescence spectra of the addition of Zn^**II**^ to a stoichiometric 1:3 Eu^**III**^:(L^**6**^)^−^ solution 16.4 μM in ligand, pH 7.4**.

Figure S5 **Stoichiometric solution 1:3 Eu^III^:(L^6^)^−^ (left) and 1:1:3 Eu^III^:Zn^II^:(L^6^)^−^ under irradiation at 366 nm; total ligand concentration: 16.4 μM in ligand, pH 7.4**.

Figure S6 **Emission spectra of solutions in Tris-HCl 0.1 M (pH 7.4) with different stoichiometries; λ_exc_ = 3.7 nm; [(L^6^)^−^]_t_ = 16.2 μM; the star denotes an artifact (2nd order Rayleigh band from excitation beam)**.
