# Supplementary material for: Self-assembly of a helical zinc-europium complex: speciation in aqueous solution and luminescence
Source: Front Chem. 2013 Sep 11;1:15. doi: 10.3389/fchem.2013.00015 (PMC3982565; doi:10.3389/fchem.2013.00015)
Supplement: Figure S1 — (Top) Re-calculated spectra from the titration of HL6 with zinc perchlorate at 295 K and pH 7.4. (Bottom) Absorbance values extracted at different wavelengths during the titration compared with theoretical prediction from the stability extracted from the fit procedure (Table 1). [file DataSheet1.PDF]

Self-assembly of a helical zinc-europium complex: speciation in aqueous solutions and luminescence

Emmanuel Deiters,[1] Svetlana V. Eliseeva,[1,2] and Jean-Claude G. Bünzli[1,3]*

[1] *Institute of Chemical Sciences and Engineering, École Polytechnique Fédérale de Lausanne, CH-1015 Lausanne, Switzerland*

[2] *Centre de Biophysique Moléculaire CNRS UPR 4301, F-45000 Orléans, France*

[3] *WCU Center for Next Generation Photovoltaic Systems, Korea University, Sejong Campus, Jochiwon-eup, Sejong-si, 339700 Republic of Korea*

Supplementary Information

6 pages

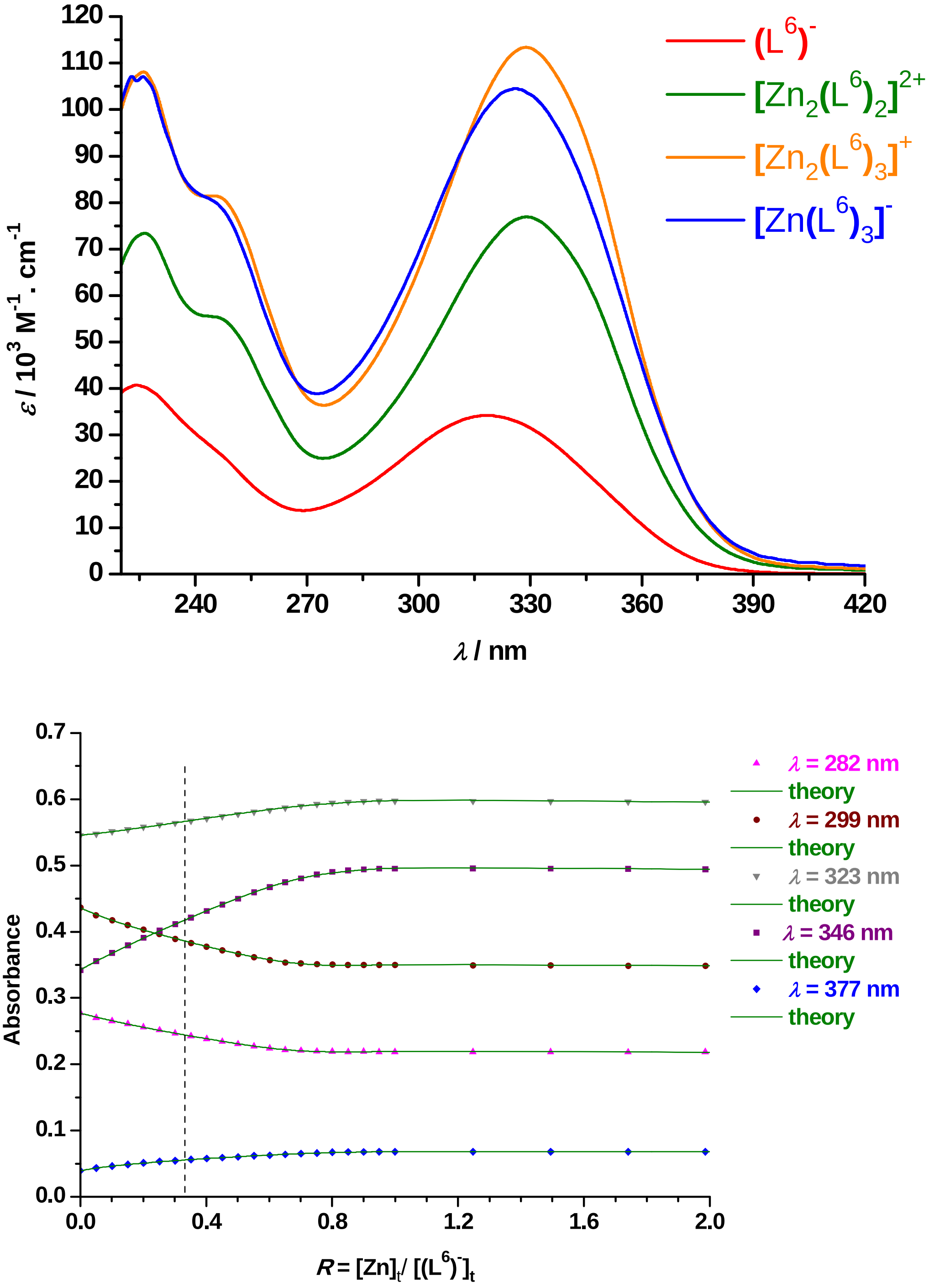

Figure S1. (Top) Re-calculated spectra from the titration of HL^6 with zinc perchlorate at 295 K and pH 7.4. (Bottom) Absorbance values extracted at different wavelengths during the titration compared with theoretical prediction from the stability extracted from the fit procedure (Table 1).

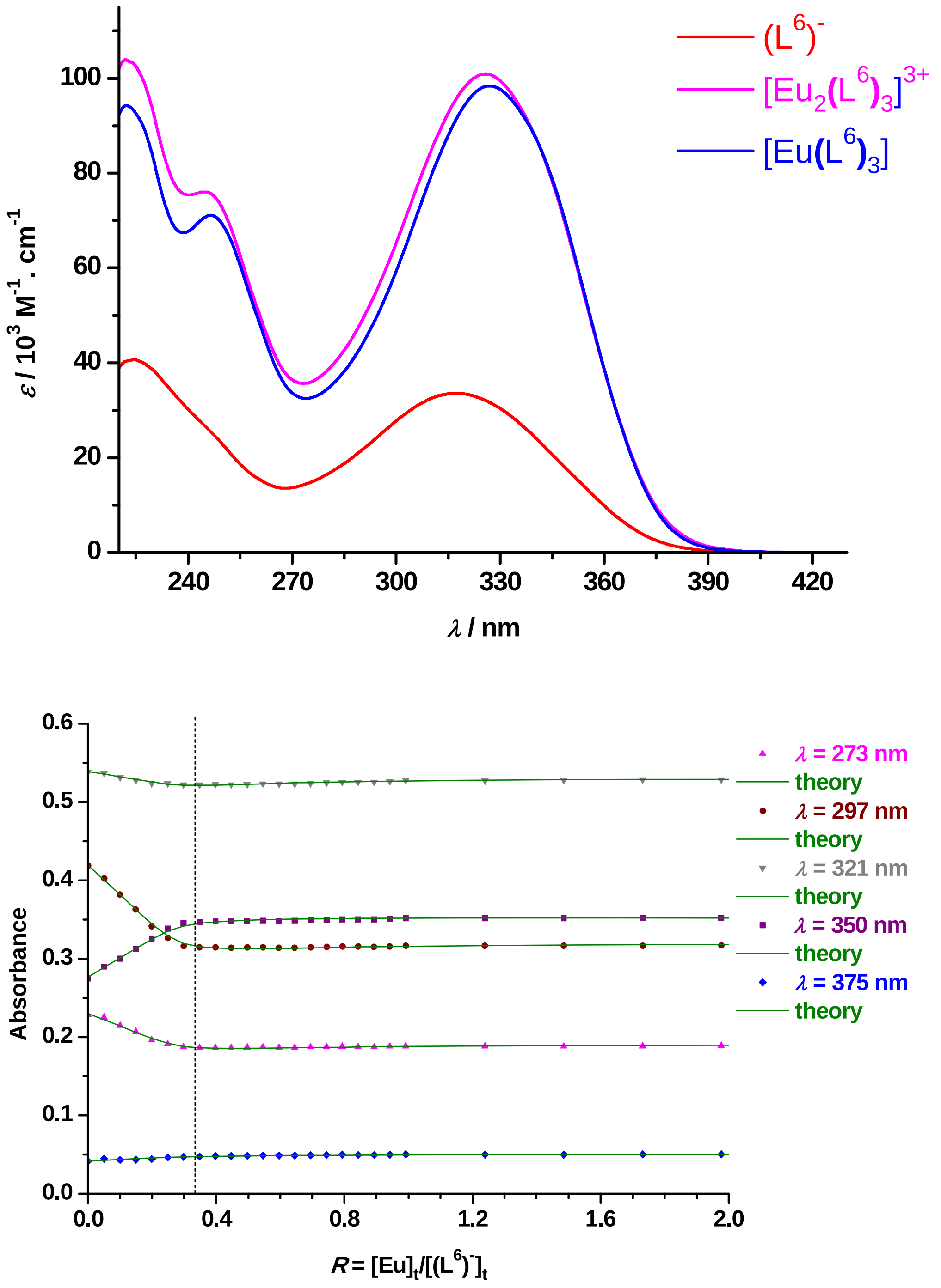

Figure S2. (Top) Re-calculated spectra from the titration of HL^6 with europium perchlorate at 295 K and pH 7.4. (Bottom) Absorbance values extracted at different wavelengths during the titration compared with theoretical prediction from the stability extracted from the fit procedure (Table 1).

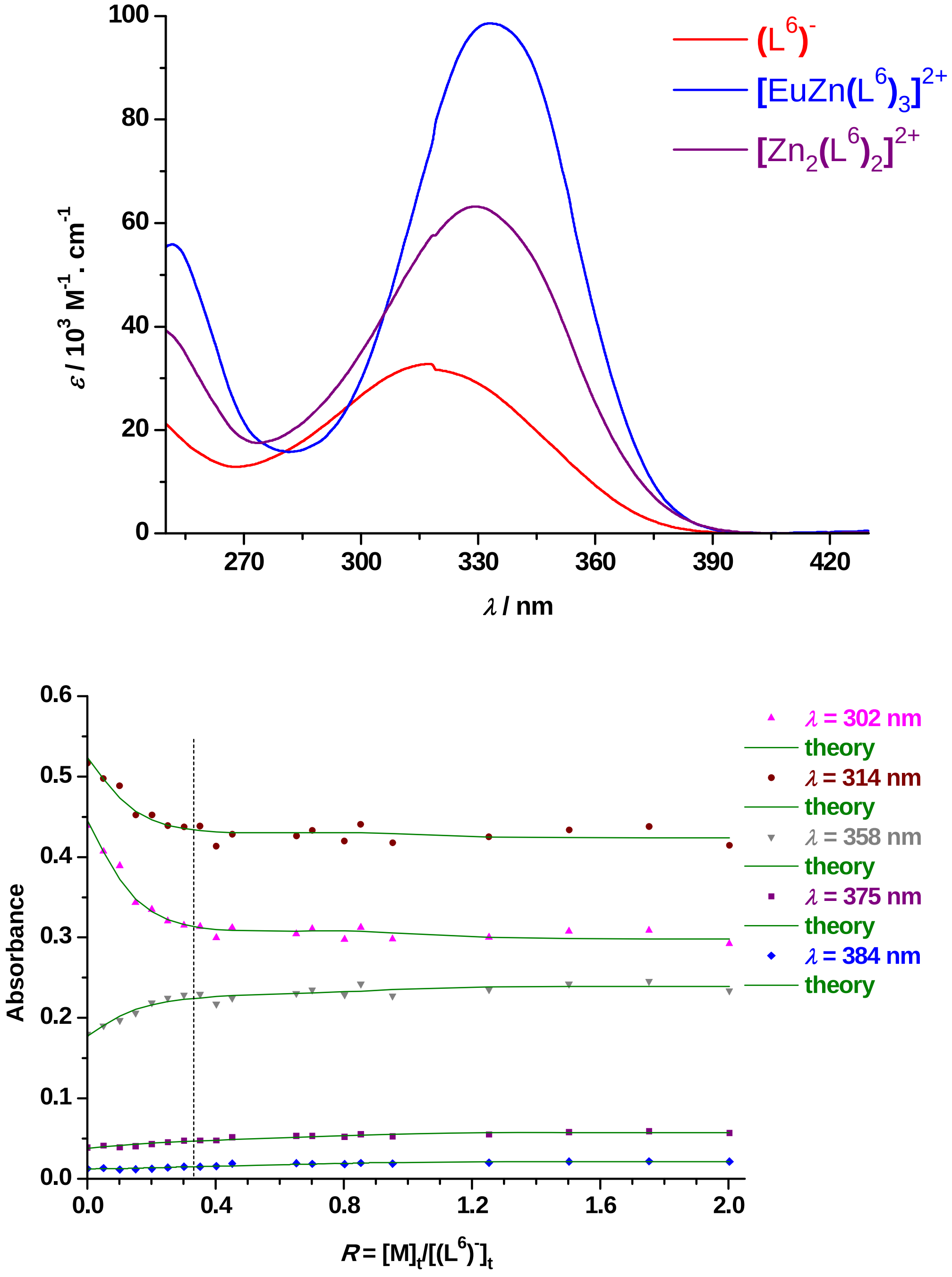

Figure S3. (Top) Re-calculated spectra from the titration of HL^6 with zinc and europium perchlorate (1:1) at 295 K and pH 7.4. (Bottom) Absorbance values extracted at different wavelengths during the titration compared with theoretical prediction from the stability extracted from the fit procedure (Table 1).

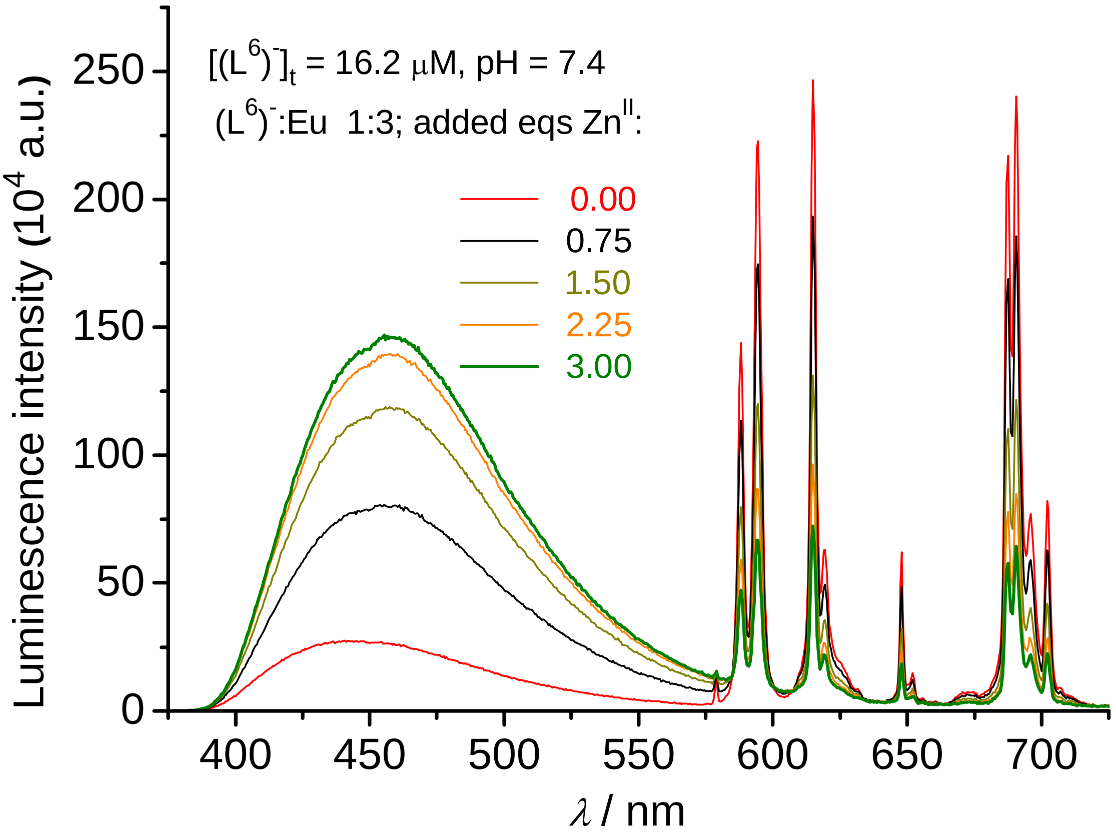

Figure S4. Effect on luminescence spectra of the addition of Zn^{II} to a stoichiometric 1:3 Eu^{III}:$(L^6)^-$ solution 16.4 µM in ligand, pH 7.4.

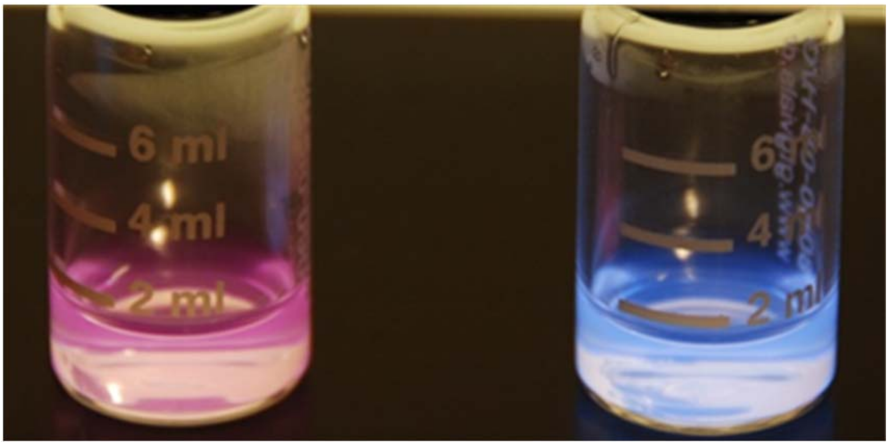

Figure S5. Stoichiometric solution 1:3 Eu^{III}:$(L^6)^-$ (left) and 1:1:3 Eu^{III}:Zn^{II}:$(L^6)^-$ under irradiation at 366 nm; total ligand concentration: 16.4 µM in ligand, pH 7.4.

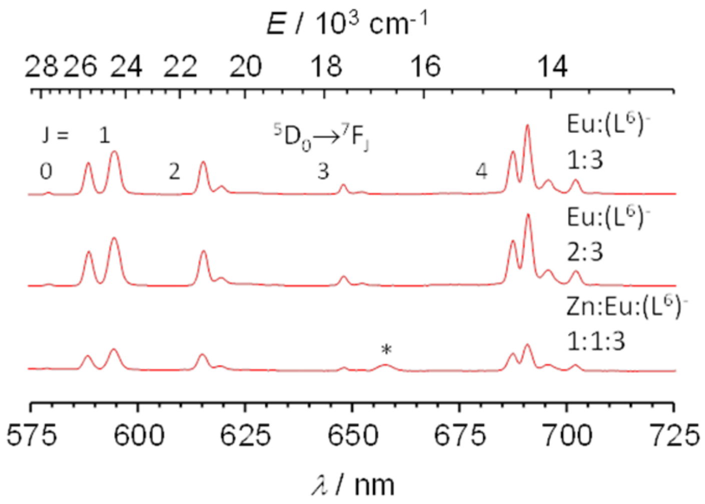

Figure S6. Emission spectra of solutions in Tris-HCl 0.1 M (pH 7.4) with different stoichiometries; λ_{exc} = 327 nm; $[(L^6)^-]_t$ = 16.2 μM; the star denotes an artefact (2nd order Rayleigh band from excitation beam).
